# Topical and transdermal lipid-polymer hybrid nanoparticles (LPN): an integration in advancing dermatological treatments

**DOI:** 10.1007/s13346-025-01940-7

**Published:** 2025-08-13

**Authors:** Kok-Hou Lok, Hooi Leong Loo, Lay-Hong Chuah

**Affiliations:** School of Pharmacy, Monash University Malaysia, Bandar Sunway, Subang Jaya, 47500 Selangor Malaysia

**Keywords:** Nanoparticles, Lipid–polymer hybrid nanoparticles, Topical treatments, Skin, Dermatological treatment and cosmetic

## Abstract

Lipid–polymer hybrid nanoparticles (LPN) are an integration or “collaboration” between the two distinct drug delivery platforms of lipid and polymeric carriers. The idea centres on coining the advantages of both materials while attempting to overcome the limitations inherent to each component, thus improving biocompatibility, drug loading, stability, size uniformity, and controlled release properties. Since their emergence over two decades ago, LPN have attracted growing interest in various therapeutic areas such as cancer, neurological disorders, osteoarthritis, and COVID-19 viral infections. Their structural diversity has expanded from the classical polymeric core-lipid shell to its inverse structure of lipid core-polymeric shell and homogeneous lipid-polymer blends, producing nine types of LPN under these structural classes. Correspondingly, preparation strategies have evolved from two-step methods to integrated one-step method of nanoprecipitation, single-emulsification-solvent evaporation, and double-emulsification-solvent evaporation in the early 2010s. More recently, novel methods such as self-assembly, modified ionic gelation, modified ethanolic injection, film rehydration, and hot-melt emulsification have been introduced, with hot-melt emulsification showing particular promise for scalability. In this context, the present review proactively introduces an updated structural classification and proposes a revision of existing formulation strategies by expanding the one-step and two-step framework to incorporate emerging methods tailored for dermatological applications. While LPN are often portrayed as a better version of lipid and polymeric-based nanoparticles, their practical applicability in dermatological treatments remains an open question. Therefore, this review evaluates LPN’s clinical and translational potential in dermatology applications such as, wounds, skin infections, dermatitis, psoriasis, skin cancer, pain management, and cosmetic applications.

**Graphical abstract:**

A collaborative fusion of lipid and polymer to create Lipid-Polymer Hybrid nanoparticles for advanced dermatological treatments

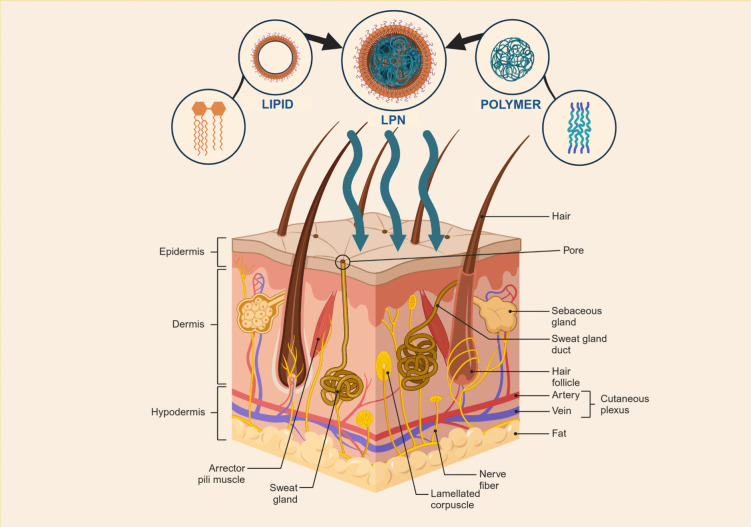

## Introduction

Dermatological diseases with over 3,000 documented varieties represent a noteworthy public health concern and reportedly affect individuals of different ages and socioeconomic backgrounds [1]. A recent survey across 27 European countries estimated that 185 million individuals aged 18 years and above experienced at least one dermatological disease within a 12-month period [1]. These dermatological diseases range from acute skin infections to chronic inflammatory psoriasis and dermatitis, many of which are highly visible and can extend their impact beyond physical symptoms to profound psychological consequences of depression, anxiety, and diminished self-esteem [2]. Among these diseases, skin cancer poses a particularly grave concern for its potential fatal outcomes. While addressing these clinical needs remains a priority, there is a constant desire from the public to improve skin quality and appearance [3, 4]. Consequently, it creates considerable market potential in this field and advances the development of novel therapeutic and cosmetic solutions from the academic and industry sectors.

Among the earliest commercialised nanoparticle-based cosmetic formulations were lipid-based nanoparticles (LBN) Niosomes® and liposomal Capture®, introduced by Lancôme and Dior in 1986, respectively [5]. Since then, leading cosmetic companies actively incorporated LBN into their formulations, and their applications have also received significant attention for clinical applications such as psoriasis and atopic dermatitis [5–7]. These nanoparticles offer key advantages including but not limited to cost-effective production, high entrapment efficacy, scalability, and a lipid composition closely resembling the skin’s [8].

Likewise, polymeric-based nanoparticles (PBN) have gained prominence in dermatological research due to their tunable physicochemical properties, controlled drug release profiles, chemical versatility, low polydispersity and high stability, positioning them as promising candidates for dermatological applications [5–7, 9, 10]. These advantages have not only captured the attention of researchers but also spurred the development and commercialisation of several PBN-based formulations in both pharmaceutical and cosmetic products [11, 12]. In pharmaceutical settings, Estrasorb*™* remains the only FDA-approved polymeric micelle formulation for topical use, delivering oestradiol transdermally in a controlled and sustained manner to manage vasomotor symptoms associated with menopause [13]. Another notable example is Tyrospheres*™*, a paclitaxel-loaded nanosphere system made from tyrosine-derived polymers, developed for treating hyperproliferative skin disorders such as psoriasis [14]. In the cosmetic industry, L’Oréal Paris’ Plénitude Revitalift cream employs polymeric nanocapsules to enhance the penetration and stability of retinol, a common anti-ageing ingredient [15].

Despite their broad applications and advantages, liposomal LBN that received significant attention in the 80 s suffer from poor stability, batch-to-batch variability, short circulation times, and limited active targeting, whereas PBN encounter challenges with scalability, high production costs, reliance on organic solvents, and low drug-loading capacity [16, 17]. To overcome these limitations, lipid-polymer hybrid nanoparticles (LPN) have emerged as a promising alternative. While LPN are often regarded as a fusion of liposomes and PBN, Wu asserted that they were initially developed in the late 1990 s and early 2000 s to enhance the loading and sustained release of water-soluble, ionic drugs within solid lipid nanoparticles [8, 18–23]. Despite these differing perspectives and the use of different lipid excipients, they show promising results in many applications, such as cancer, neurological disorders, COVID-19 treatment and osteoarthritis [18–22].

In parallel with these developments, an increasing number of studies have reported on the use of LPN in dermatological applications, including wound healing, infections, dermatitis, psoriasis, skin cancer, pain management, and cosmetics. This increment reflects growing academic attention toward LPN for skin-associated conditions. However, to the best of our knowledge, no review has systematically examined both topical and transdermal LPN formulations within this specific therapeutic area. Guided by key search terms related to LPN, a broad range of dermatological clinical conditions, and cosmetics across databases including PubMed, Scopus, Web of Science, MEDLINE, EMBASE, and Google Scholar, this review aims to consolidate the existing evidence, evaluate reported outcomes, and assess any progress toward clinical or industrial translation. It also introduces the newer classification of LPN structures and revises formulation methods recently reported for dermatological use, particularly by expanding the current one-step and two-step framework to incorporate newer techniques. In addition to their established benefits in enhancing drug loading, controlled release and safety profiles, LPN hold particular relevance in dermatology due to the skin-compatible properties of lipid components that may improve absorption and the additional therapeutic potential of certain polymers used in their design. This combination of therapeutic and delivery attributes positions LPN as a platform with significant potential, encouraging further investigation, discussion and wider application in dermatological research.

## Types, characteristics and compositions of lipid-polymer hybrid nanoparticles

**Fig. 1:**
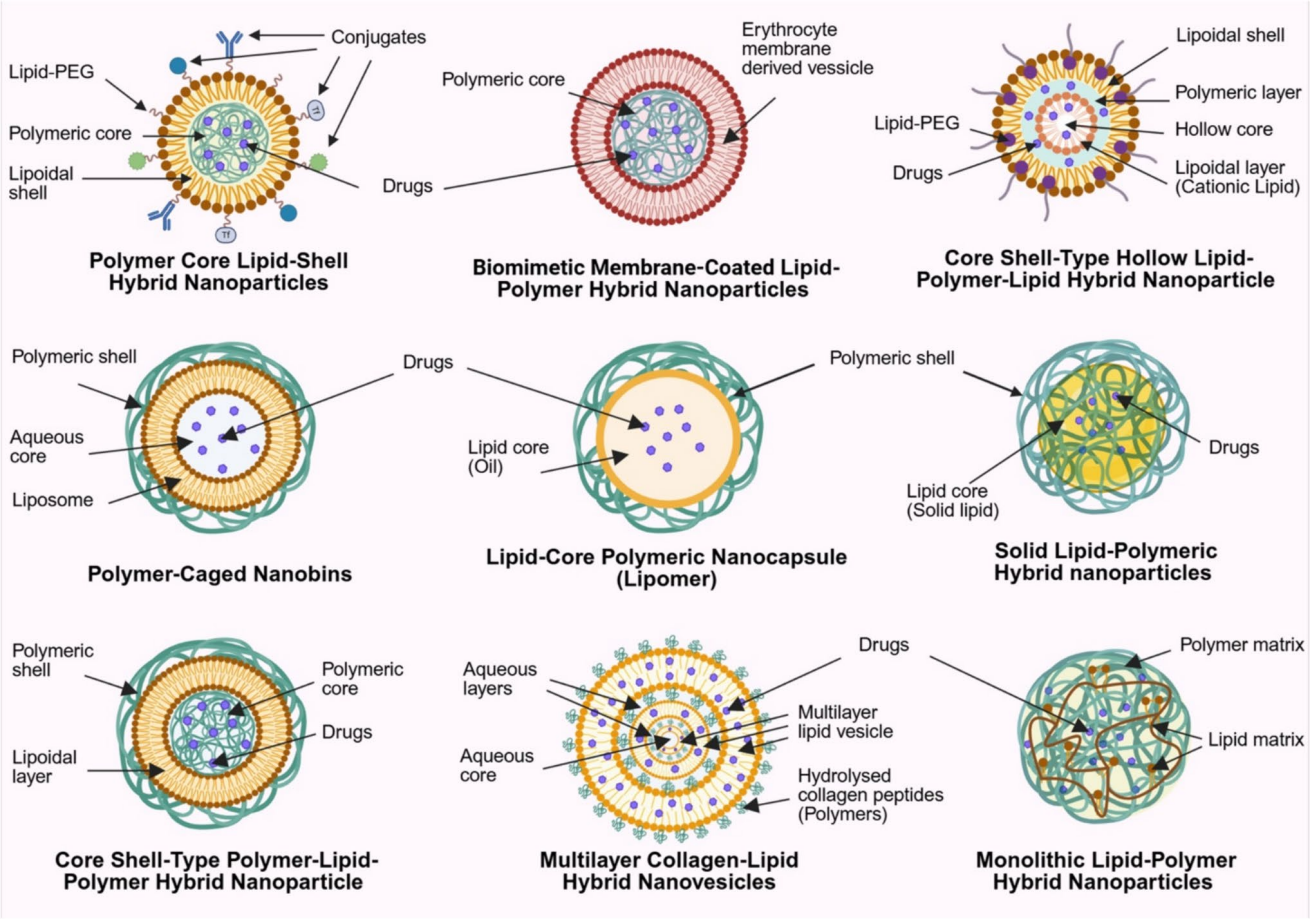
Structural configurations of various types of lipid-polymer hybrid nanoparticles (LPN), including polymeric core–lipid shell hybrid nanoparticles (PCLSHN), biomimetic membrane-coated LPN, core–shell-type hollow lipid-polymeric-lipid hybrid nanoparticles (CSTHLPLHN), polymeric-cage nanobins, lipid-core polymeric nanocapsules (Lipomer), solid LPN, core–shell-type polymer-lipid-polymer hybrid nanoparticles (CST-PLP-HN), multilayered collagen-lipid hybrid nanovesicles, and monolithic LPN. Abbreviations are shown alongside each configuration in the figure for ease of reference

The drug delivery functionality of LPN lies in their modular architecture, which integrates lipid and polymer components to form hybrid systems with tunable physicochemical properties. Each component contributes distinct characteristics that can be strategically modulated to achieve specific drug delivery behaviours or therapeutic outcomes. Polymers generally offer excellent mechanical strength, controlled drug release, and potential responsiveness to environmental stimuli, whereas lipids enhance encapsulation efficiency and biocompatibility, with the added potential for functionalisation with targeting moieties. Notably, lipid components are particularly advantageous for dermatological applications. Their interaction with the stratum corneum (SC) enhances membrane fluidity, disrupts the highly ordered lipid bilayers, and promotes skin hydration, ultimately increasing permeability and facilitating more effective delivery of therapeutic agents into deeper dermal layers. Moreover, the vast array of polymeric and lipidic categories provides extensive opportunities for formulation customisation. Each class exhibits unique physicochemical traits, enabling researchers to combine components in a modular fashion similar to assembling "Lego bricks". This flexibility applies not only to material selection but also to the architectural design of the system, as illustrated in Fig. 1, allowing precise tailoring of LPN for specific functional and therapeutic outcomes.

The most straightforward LPN design is polymeric core–lipid shell hybrid nanoparticles (PCLSHN), where natural or synthetic polymers such as chitosan, hyaluronic acid, polylactic-co-glycolic acid (PLGA), and polycaprolactone (PCL) form a drug-loaded polymeric core that enables controlled release. In contrast, the surrounding lipid layer can be strategically engineered as a mono- or bilayer to modulate the release profile and provide an additional diffusional barrier that attenuates burst release while protecting the encapsulated drug from degradation [8]. (Fig. 1) Together with the slow degradation kinetics of the polymeric core, this configuration offers enhanced sustained release performance compared to conventional nanoparticles and enables fine-tuning of drug delivery characteristics through rational material selection and architectural design. The lipid layer can be derived from solid lipids, fatty acids, cholesterol, and phospholipids, many of which are biological membranes, sebum and skin components [8, 24]. Their incorporation into LPN enhances its resemblance to the skin, improving drug permeation and biocompatibility for dermatological applications, while phospholipid-based excipients may further act as surfactants to stabilise the formulation [25–27].

However, the instability of natural lipids has led to the development of synthetic alternatives such as polyethylene glycol (PEG)-ylated phospholipids and 1,2-diacyl-P-O-ethylphosphatidylcholine [8]. While PEGylation is commonly included as an outermost third layer in PCLSHN to extend systemic circulation time and provide steric repulsion for better stability, this structural modification has not been widely adopted in LPN for dermatological applications in recent years [27]. The limited adoption may be due to inconsistent findings regarding its impact on dermatological drug delivery. For instance, Rangsimawong et al. [28] reported that PEGylation affects skin hydration differently depending on the type of LBN, while another study [29] suggests that PEGylation on PBN enhances drug permeation in intact skin but not impaired skin. Such variability and the need to treat various dermatological conditions involving impaired skin integrity may explain the reduced suitability of PEGylation for this application.

Beyond lipids and polymers, surfactants are often required to provide additional structural support and stability for LPN made with phospholipids [27]. Their inclusion is also particularly beneficial for transdermal drug delivery, as surfactants facilitate SC fluidisation and enhance drug permeation [30]. This unique structural composition underscores the advantages of hybrid systems in integrating the benefits of LBN, which are absent in PBN formulations. Furthermore, LPN can be functionalised by targeting moieties such as antibodies, transferrin, folic acid and other bioactive molecules to enhance selectivity for conditions like psoriasis and skin cancer, enabling more precise drug delivery [8]. (Fig. 1).

The modularity of LPN, with its diverse combinations of polymers, lipids, lipid-PEG, surfactants, and functional conjugates, provides extensive formulation flexibility. This versatility enables the broad customisation of PCLSHN to achieve optimal drug delivery characteristics for specific therapeutic needs and facilitates the development of entirely new LPN architectures (Fig. 1). One such advancement is the integration of biological membranes to create biomimetic membrane-coated LPN. Hu et al. [31] were among the early groups to replace the outer lipid layer of LPN with red blood cell (RBC) membranes, demonstrating superior retention after 24 h (29%) compared to LPN enveloped by lipid–PEG layer (11%). This invention is particularly interesting from an application and translational perspective. On the one hand, it highlights the potential of LPN in personalised medicine; on the other, the distinct antigens on RBC surfaces may trigger immunogenic responses upon transfusion, limiting their applicability to the population with different blood types, giving two contrasting implications. Nevertheless, such LPN are unlikely to be relevant for dermatological applications. In addition to the laborious extraction and complex formulation process, the use of red blood cell membranes is biologically irrelevant to skin tissue and offers limited value in transdermal or topical delivery. Instead, a wide range of well-characterised natural lipid excipients that mimic the composition and behaviour of skin lipids already exist and provide more practical, scalable, and biologically appropriate options for enhancing dermal drug delivery.

Beyond biomimetic strategies, another challenge associated with polymeric core-type LPN is their limited capacity for encapsulating hydrophilic drugs [19]. One approach to overcoming this challenge is the development of core–shell-type hollow lipid-polymeric-lipid hybrid nanoparticles (CSTHLPLHN), which consist of an inner aqueous core enclosed by a hydrophobic polymeric layer and further surrounded by an outer lipid-PEG layer. This design enables the co-loading of hydrophilic and hydrophobic small-drug molecules or even siRNA, making it particularly advantageous for overcoming multidrug resistance in malignancies [19].

The LPN discussed thus far primarily feature a lipid layer as the outermost shell. However, two additional configurations can be broadly classified: (1) systems where a polymer serves as the outer shell instead of a lipid layer and (2) blended lipid-polymer hybrid systems, where lipids and polymers are homogeneously integrated rather than arranged in distinct layers. In the first category, polymeric-cage nanobins consist of a liposomal aqueous core encapsulated within a polymeric shell to offer better structural stability and reduce premature drug leakage [19]. This structural modification enables the use of functional polymers that respond to environmental stimuli, particularly pH variations [32]. For instance, incorporating PEG or designing cross-linked polymer networks with pH-sensitive properties can allow these systems to release their payload selectively in acidic microenvironments of the tumour interstitium and intracellular vesicles [33]. These features are especially valuable in skin cancers, where the acidic tumour microenvironment may be exploited to achieve greater therapeutic precision and efficacy. However, the absence of a lipid layer at the outmost later may result in lower cellular uptake due to rapid recognition and clearance by the reticuloendothelial system (RES) [19]. Other analogous systems include lipid-core polymeric nanocapsules (Lipomer) and solid LPN, which incorporate an oil-based core and a solid-lipid core, respectively, instead of an aqueous core to facilitate the encapsulation of lipophilic drugs [34–38].

Khan et al. [39] recently reported the development of an LPN consisting of a polymeric drug-loaded core surrounded by a lipid layer and followed by an additional outer polymeric shell. However, the authors neither provide empirical validation nor a formal classification for their product. Based on the given structural description, this design appears to be another multilayer LPN variant with an inverse configuration of CSTHLPLHN, forming what can be described as core–shell-type polymer-lipid-polymer hybrid nanoparticles (CST-PLP-HN). Similarly, another polymeric shell-based multilayer LPN was recently developed by Rahman et al. [40–43] but with structural validation. Their work introduced hydrolysed collagen peptides (HCP) as non-covalent crosslinkers to construct highly robust multilayered collagen-lipid hybrid nanovesicles, where each lipid layer is coated with the HCP polymeric network that bridges to the subsequent lipid layer in a repeating sequence, culminating in an outermost HCP layer at the surface. This hierarchical assembly with approximately 7 nm thickness of lipid layers and aqueous layers containing the collagen network at around 4 nm provides a novel strategy to enhance the structural stability of the LPN and maintain its biocompatibility.

Contrary to the aforementioned systems where either a lipid or polymer constitutes the outermost shell of the LPN, monolithic LPN is characterised by the randomly scattered solid and liquid lipid molecules within a hydrophobic polymeric matrix [19, 39, 44, 45]. The resulting colloidal architecture provides a thermodynamically stable environment that enhances the encapsulation efficiency of hydrophobic and lipophilic drugs while enabling controlled and sustained release profiles. Collectively, these LPN systems underscore the structural complexity and functional versatility of the platform, which arises from the wide variety of lipids, polymers, and surfactants employed in their design. Table 1 summarises these LPN types along with their key formulation components and representative examples, drawing from both the current scope of discussion and relevant literature [8, 23, 46].

**Table 1:** Classification of LPN types with corresponding main components of polymer, lipid and surfactants examples

**LPN Types**	**Main Components**		**Examples**
Polymer Core Lipid-Shell Hybrid Nanoparticles (PCLSHN) & Core Shell-Type Hollow Lipid-Polymer-Lipid Hybrid Nanoparticles (CSTHLPLHN)	Polymer	Natural	· Albumin · Alginic acid · Chitosan and its derivatives · Dextran · Gelatin · Heparin · Hyaluronic acid · Protamine sulphate · Sodium alginate
		Semi-synthetic	· Ethyl cellulose · HPOE (hydrolyzed polymer of epoxidized soybean oil) · N-trimethyl chitosan · Poly(methcrylic acid) · Starch grafted tween-80 · Xelorex™ RS 1100
		Synthetic	· Eudragit · Poloxamer · Polyacrylic acid · Poly(allylamine hydrochloride) · Polyamidoamine (PAMAM) · Polycaprolactone (PCL) · Polyethleneimine (PEI) · Polyethylene glycol (PEG) · Poly (lactic acid) (PLA) · Poly(lactic-co-gylcolic acid) (PLGA) · Poly(N-isopropylacrylamide) (PNIPAm) · Polyvinylamine · Poly(2-hydroxyethyl methacrylate) (PHEMA)
	Lipid	Phospholipid	· 1,2-dioleoyl-sn-glycero-3-phosphoethanolamine (DOPE) · 1,2-dioleoyl-sn-glycero-3-phosphocholine (DOPC) · 1,2-Distearoyl-sn-glycero-3-phosphoethanolamine (DSPE) · 1,2-distearoyl-sn-glycero-3-phosphocholine (DSPC) · Phosphatidylcholine · Soybean lecithin (Phospholipon 90 G)
		Cationic lipid	· N[1-(2,3dioleyloxy) propyl]-N,N,N trimethylammonium chloride (DOTMA) · 1,2-dioleoyl-3-trimethylammonium propane (DOTAP) · Dioctadecylamidoglycylspermine (DOGS) · 1,2-dipalmitoyl-3-trimethylammonium-propane (DPTAP) · [N-(N′,N′-dimethylaminoethane)-carbamoyl]cholesterol (DC-Chol)
		PEG-lipid	· DOPE-PEG2000 · DSPE-PEG2000 · DSPE-PEG5000
		Sterol	· Cholesterol
		Saturated fatty acid	· Capric acid (C10) · Lauric acid (C12) · Myristic acid (C14) · Palmitic acid (C16) · Stearic acid (C18) and its sodium salt · Behenic acid (C22)
		Unsaturated fatty acid	· Oleic acid (C18) · Docosahexanoic acid (C22) · Linoleic acid
		Solid lipid, hard fat and glyceride	· Glyceryl behenate (Compritol®888 ATO) · Glyceryl behenate (mono) · Glyceryl palmitostearate (Precirolol®ATO 5) · Glyceryl monostearate (Imvitor®900) · Witepsol®
	Surfactants	Non-ionic	· D-α-tocopheryl polyethylene glycol succinate (TPGS) · Polysorbate-20 · Polysorbate-60 · Polysorbate-80 · Polyvinyl alcohol (PVA) · Sorbitan monooleate · Sorbitan monostearate
		Ionic	· Cetyltrimthylammonium bromide · Dioctylsodium sulfosuccinate · Sodium cholate · Sodium glycocholate · Taurocholic acid sodium salt
		Zwitter ionic	· Egg lecithin (Lipoid®E 80) · Phosphatidylcholine · Soyabean lecithin (Lipoid® S 75, S 100)
		Non-ionic block copolymers	· PEO-PPO-PEO (poloxamer 182, 188, 407) · Poloxamine
Biomimetic Membrane-Coated Lipid-Polymer Hybrid Nanoparticles	Polymer	Synthetic	· Poly(lactic-co-gylcolic acid) (PLGA)
	Lipid	Cell-derivative lipids	· Red blood cell membrane
Polymer-Cages Nanobins	Polymer	Natural	· Chitosan
	Lipid	Phospholipid	· Lecithin · Phosal® 53 MCT
	Surfactant	Non-ionic	· Tween 80
Lipid-Core Polymeric Nanocapsules (Lipomer)	Polymer	Synthetic	· Poloxamer 188 · Ethyl cellulose
	Lipid	Liquid lipid (Oil)	· Maisine 35–1 · Medium chain triglycerides
	Surfactant	Non-ionic	· Tween 80 · Span 60
Solid Lipid-Polymeric Hybrid Nanoparticles	Polymer	Synthetic	· Polyethleneimine (PEI) · Poly(allylamine hydrochloride)
	Lipid	Solid lipid, hard fat and glyceride	· Crodamol SS™ · Compritol® 888 ATO
	Surfactant	Non-ionic block copolymers	· Poloxamer 188
Core Shell-Type Polymer-Lipid-Polymer Hybrid Nanoparticles (CST-PLP-HN)	Polymer	Natural	· Chitosan
	Lipid	Phospholipid	· Lecithin
	Surfactant	Non-ionic	· Tween 80
Multilayer Collagen-Lipid Hybrid Nanovesicles	Polymer	Natural	· Hydrolysed collagen peptides (HCP)
	Lipid	Cationic lipid	· 1,2-dioleoyl-3-trimethylammonium propane (DOTAP)
Monolithic Lipid-Polymer Hybrid Nanoparticles	Polymer	Synthetic	· Di-block copolymer of DL-lactide and Methoxy poly(ethylene glycol)
	Lipid	Solid lipid, hard fat and glyceride	· Precirol® ATO 5
		Liquid lipid (oil)	· Linoleic acid
	Surfactant	Non-ionic surfactant	· Tween 80

## Formulation methods of lipid-polymer hybrid nanoparticles

**Fig. 2:**
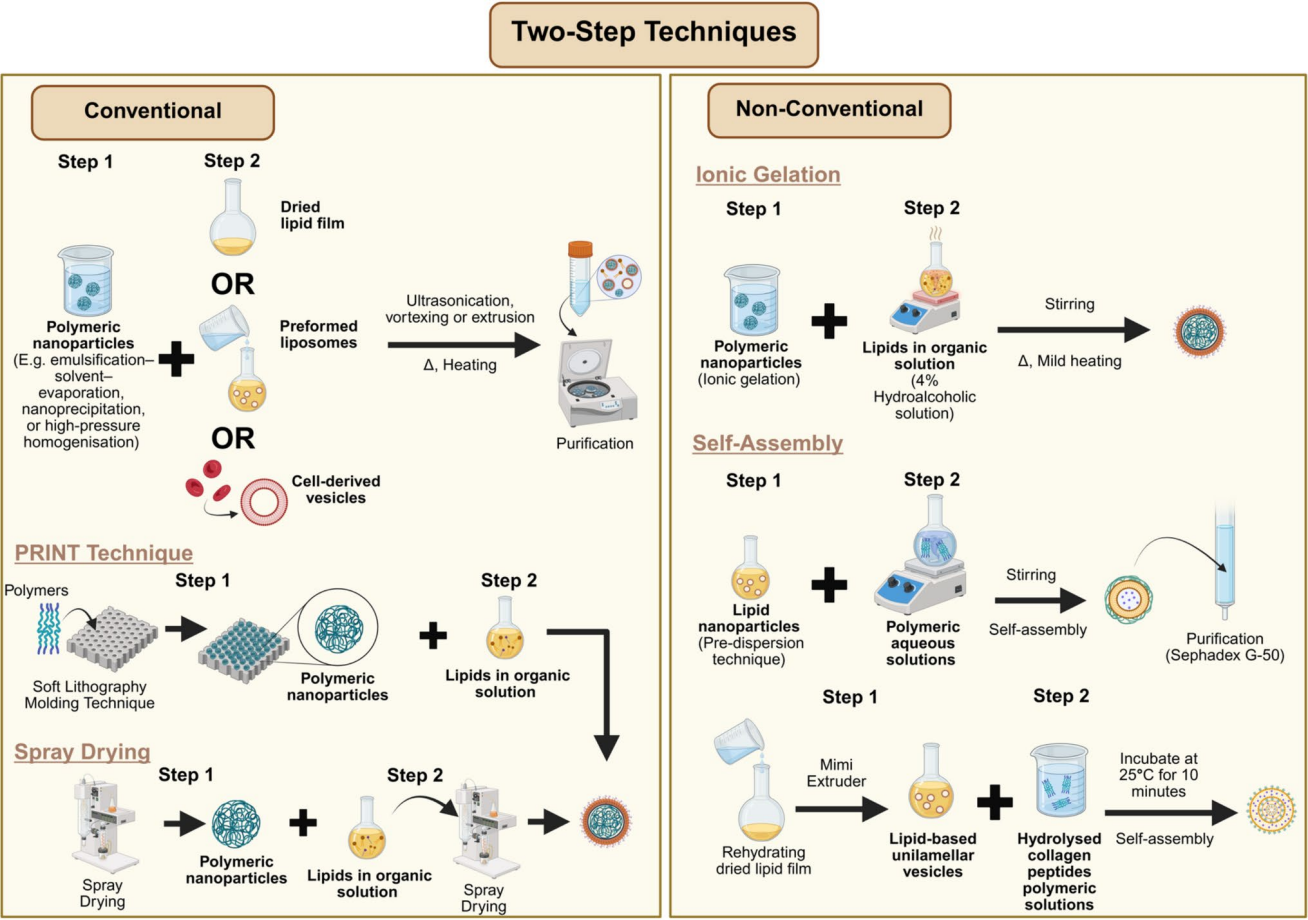
Conventional and non-conventional two-step techniques for formulating LPN

**Fig. 3:**
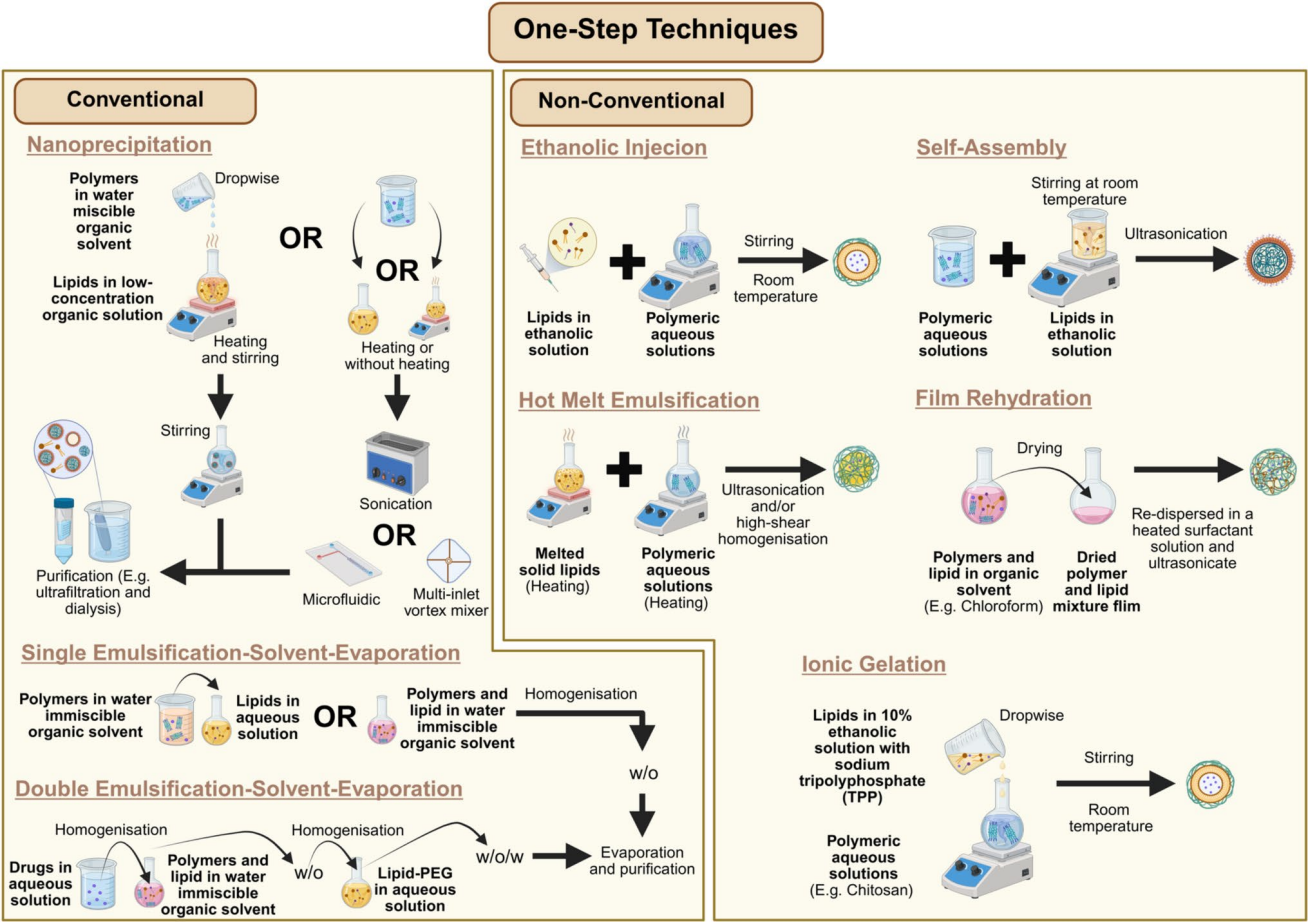
Conventional and non-conventional one-step techniques for formulating LPN

The formulation methodologies of LPN have undergone continuous refinement, modification, and exploration by the research community, making this aspect of the field both dynamic and fundamental. Historically, LPN formulation was primarily built upon four main methods in the early 2010s: (1) two-step methods, (2) one-step nanoprecipitation, (3) one-step single emulsification–solvent–evaporation (ESE), and (4) one-step double ESE, which represented the foundational techniques in this domain [47, 48]. While these were broadly categorised into two-step and one-step methods, Hadinoto and colleagues were among the earliest to introduce an additional classification by distinguishing between conventional and non-conventional methods in their 2013 review, a framework that remains widely accepted and referenced recently [22, 46, 48–51]. However, despite the emergence of various novel formulation strategies over the past decade, most recent review articles focus solely on these methods without exploring newer alternatives [49, 52]. An exception is the review by Gajbhiye et al. [19] which investigated alternative techniques for developing different types of LPN. Nonetheless, the review did not specify the formulation names or explicitly classify the methods as two- or one-step. Whether this omission of including and categorising newer formulation methods in these recent reviews stems from the inherent ambiguity in classification or other considerations remains unclear. This lack of detailed discussion on emerging techniques may partly explain the limited recognition and adoption of LPN. Hence, while this work adheres to the nomenclature used by the original authors when discussing recent works, it also proactively categorise the newer reported methods into two- or one-step, recognising and respects the foundational contribution of Hadinoto et al. [48]. At the same time, it proposes a revision to the conventional and non-conventional classification to better reflect the field’s development over the past decade, particularly by incorporating, categorising and comparing newer methods within dermatological applications, as summarised in Fig. 2 and 3, Table 2, and the section below.

**Table 2:** A comparative review of one-step and two-step conventional and non-conventional methods for lipid–polymer hybrid

**Formulation Methods**		**LPN Type Produced**	**Advantages**		**Disadvantages**	
			General*	Specific**	General*	Specific**
**Two-Step**						
Conventional	Nanoprecipitation	Polymer Core Lipid-Shell Hybrid Nanoparticles	· Particle size and drug loading of the core can be more precisely controlled. · Theoretical amount of the lipid required to uniformly coat the core with a uniform bilayer of phospholipids can be calculated based on the properties of the core and phospholipids.		· Time consumption and laborious · High cost of production · Limited capacity to encapsulate hydrophilic drugs · Generally difficult to scale up · Generally require additional purification process	· Involve organic solvent
		Biomimetic Membrane-Coated Lipid-Polymer Hybrid Nanoparticles				· High-energy consumption when extracting red blood cell membrane · Involve organic solvent
	Emulsification–solvent evaporation	Polymer Core Lipid-Shell Hybrid Nanoparticles				· Involve organic solvent · Thermal-based method and not ideal for encapsulating heat-sensitive drugs
	High-pressure homogenisation					· High-energy consumption method ·
	Spray drying					· Involve hazardous organic solvent like dichloromethane · Thermal-based method and not ideal for encapsulating heat-sensitive drugs · Large particle size
	Soft lithography-based technique or Particle Replication in Nonwetting Templates (PRINT)			· Offers precise control over PCLSHN’s particle size and morphology · Scalable potential · High reproducibility		· Involve organic solvent · Thermal-based method and not ideal for encapsulating heat-sensitive drugs
Non-conventional	Ionic gelation	Polymer Core Lipid-Shell Hybrid Nanoparticles		· Low-energy based mixing (stirring) · Low concentration (4%) hydroalcoholic solvent · Gentle heating at 37 °C		· Involve organic solvent
	Self-assembly	Polymer-caged nanobins		· Heating-free method · Solvent-free method · Low-energy mixing (stirring)		
		Multilayer Collagen-Lipid Hybrid Nanovesicles		· Gentle heating at 37 °C to produce lipidic core · Simple mixing and incubation of lipidic core and polymeric solution at 25˚C for 10 minutes		· Involve organic solvent · Extremely lengthy process to obtain purified polymers (7 days)
**One-Step**						
Conventional	Nanoprecipitation	Polymer Core Lipid-Shell Hybrid Nanoparticles	· More streamlined and simplified formulation method · More affordable · Generally fast formulation process	· Low-energy mixing (stirring) · Prove to obtain small and uniform size when using bath sonication or microfluidic · Scalable potential when using bath sonication or microfluidic	· Generally require additional purification process	· Limited to encapsulate drugs soluble in water-miscible organic solvents · Involve organic solvent · Thermal-based method and not ideal for encapsulating heat-sensitive drugs · High-energy consumption if replace simple stirring with bath sonication
	Single emulsification–solvent evaporation	Polymer Core Lipid-Shell Hybrid Nanoparticles & Lipid-Core Polymeric Nanocapsules (Lipomer)		· Specially design to encapsulate drugs soluble in water-immiscible organic solvents (hydrophobic drugs) · Heating-free method		· High-energy consumption method · Involve hazardous organic solvent · Lengthy solvent evaporation method
	Double emulsification–solvent evaporation	Core Shell-Type Hollow Lipid-Polymer-Lipid Hybrid Nanoparticles		· Enabling the encapsulation of hydrophilic and hydrophobic drugs · Heating-free method		· High-energy consumption method · Involve hazardous organic solvent · Lengthy solvent evaporation
Non-conventional	Ethanolic injection	Polymer-Cages Nanobins		· Low-energy mixing (stirring) · Heating-free method		· High-energy consumption method · Involve organic solvent
	Self-assembly	Polymer Core Lipid-Shell Hybrid Nanoparticles		· Heating-free method		· Involve organic solvent
	Ionic gelation	Core Shell-Type Polymer-Lipid-Polymer Hybrid Nanoparticles				· High-energy consumption method · Thermal-based method and not ideal for encapsulating heat-sensitive drugs · Involve organic solvent
		Polymer-caged nanobins		· Low-energy mixing (stirring) · No heating · Low concentration (10%) ethanolic solvent		· Require additional cross linker compounds such as polyanionic sodium tripolyphosphate (TPP) · Involve organic solvent
	Hot melt emulsification	Solid Lipid-Polymeric Hybrid Nanoparticles		· Does not involve organic solvent		· High-energy consumption method · Thermal-based method and not ideal for encapsulating heat-sensitive drugs
	Film rehydration	Monolithic Lipid-Polymer Hybrid Nanoparticles				· High-energy consumption method · Thermal-based method and not ideal for encapsulating heat-sensitive drugs · Involve hazardous organic solvent

Superscripts:

* General advantages and disadvantages associated with two-step and one-step formulation methods

** Specific advantages and disadvantages pertaining to the individual subtypes under the two-step and one-step formulation approaches

### Two-step approaches

#### Conventional

The two-step approach represents one of the earliest strategies for LPN formulation, which involves the independent preparation of the polymeric nanoparticle core and the lipid phase prior to their assembly. The polymeric core is typically generated via methods such as emulsification–solvent evaporation, nanoprecipitation, or high-pressure homogenisation [53–55], whereas the lipid component is commonly prepared as a dried thin film or reconstituted into vesicles through hydration of the film [56]. Upon combining the two phases, the resulting polymer–lipid suspension undergoes homogenisation (e.g., vortexing or ultrasonication) or extrusion through a porous membrane, with both processes conducted at temperatures exceeding the lipid’s gel-to-liquid crystalline transition point [31, 54–58]. This step facilitates lipid adsorption onto the nanoparticle surface, primarily through electrostatic interactions, thereby yielding constructs such as PCLSHN and biomimetic membrane-coated LPN. A subsequent purification step involving centrifugation or dialysis is often employed to eliminate unreacted lipid material [54, 56, 57].

Alternatively, the two-step approach may involve reacting pre-formed polymeric nanoparticles directly with lipid-containing solutions to produce PCLSHN. For example, polymeric cores generated via spray drying can be dispersed in a lipid-containing dichloromethane solution, followed by a second spray-drying cycle to remove the solvent and yield PCLSHN [59]. Likewise, the soft lithography-based technique, known as Particle Replication in Nonwetting Templates (PRINT), employs a mould-based process whereby a polymer solution is casted and thermally shaped into nanoscale cavities before being exposed to an aqueous lipid solution. This exposure facilitates lipid deposition onto the polymeric cores as the underlying support layer dissolves. Consequently, the PRINT method offers precise control over PCLSHN’s particle size and morphology, guided by the predefined geometry of the mould design [60].

#### Non-conventional

Recent advancements in the two-step assembly of LPN have introduced alternative strategies to form the polymeric core for dermatological applications. One such method involves utilising ionic interactions, particularly the ionic gelation of chitosan. In this approach, Hazari et al. [61] synthesised drug-loaded chitosan nanoparticles through ionic gelation and subsequently incorporated them into a lipid-containing hydroalcoholic solution (4%) under mild heating with continuous stirring to produce PCLSHN.

In contrast to polymer-core-first approaches, certain studies have reversed the self-assembly sequence by initially constructing the lipid core prior to subsequent polymer incorporation. For example, Castro et al. [62] generated liposomes using the pre-dispersion technique and gradually introduced an aqueous polymeric solution under continuous stirring for two hours to allow uniform polymer coating on the lipid core’s surface. The resulting polymer-caged nanobins were subsequently purified using Sephadex G-50 column chromatography. Likewise, a comparable structure of multilamellar nanovesicles LPN was fabricated using the self-assembly method that involves the initial preparation of lipid vesicles, wherein lipid-based unilamellar vesicles were initially prepared by rehydrating a dried lipid film and processing it through a mini extruder [40]. These preformed vesicles were then mixed with purified HCP and incubated at 25 °C for 10 min to facilitate the formation of multilamellar nanovesicles [40].

### One-step approaches

#### Conventional

Despite its utility, the two-step method presents notable inefficiencies in the formulation procedure that require more time and higher energy demands to produce polymeric nanoparticles and lipid phase (lipid film, lipid vesicle or lipid solution) before combining. In response to these limitations, early developments in LPN research introduced a more streamlined one-step approach explicitly designed to simplify this conventional two-step approach. These methods involve mixing polymer and lipid solutions directly through nanoprecipitation or ESE to facilitate simultaneous lipid-polymeric integration and nanoparticle formation.

In the nanoprecipitation, the method begins with preparing polymeric and lipid solutions separately, where the polymer and API are dissolved in a water-miscible organic solvent and lipids dispersed in heated low-concentration (4 wt %) ethanol aqueous solution [63, 64]. Accordingly, the prepared polymer mixture is added dropwise to the lipid dispersion under continuous stirring to precipitate polymers and drive lipid assembly around the precipitated polymeric core via hydrophobic interaction. The process then concludes with ultrafiltration to purify and harvest the PCLSHN.

Following the introduction of more streamlined nanoprecipitation approaches, subsequent innovations in the early years aimed to optimise the process by addressing challenges in particle uniformity, energy input, and scalability. For instance, Fang et al. [65] substituted the basic stirring step with bath sonication to deliver a more uniform energy distribution, which accelerated nanoparticle formation, reduced organic solvent usage, and improved overall productivity by 20-fold. Similarly, Valencia et al. [66] employed a microfluidic platform to refine the mixing parameters, resulting in improved particle size homogeneity and more consistent lipid coverage. To further address the limited throughput of microfluidic systems, later modifications introduced larger microchannels (Reynolds number ≈ 75, 2000 µm height) and a multi-inlet vortex reactor with four radially symmetric inlets (diameter 1100 µm), offering better microscale mixing and improved scalability for translational applications [67, 68].

Despite its advantages, the nanoprecipitation method limits the ability of LPN encapsulating API soluble in water-miscible organic solvents. To address this constraint, a one-step single ESE (oil-in-water, o/w) method using water-immiscible organic solvents has been developed to broaden the applicability of LPN for delivering diverse API [48, 69]. This approach involves dissolving the polymer and hydrophobic API in an organic solvent before mixing with an aqueous lipid solution under continuous agitation, such as homogenisation or sonication [69, 70]. Alternatively, lipids may also be dissolved in the organic phase before mixing [70]. The resulting emulsion undergoes magnetic stirring overnight at room temperature to facilitate organic solvent evaporation, followed by centrifugal purification [69, 70]. While this method has been successfully employed in developing PCLSHN and Lipomer-based LPN to encapsulate hydrophobic API, it remains unsuitable for hydrophilic API insoluble in any organic solvent. To overcome this limitation, Cheow and Hadinoto [47] introduced a double ESE approach, leveraging the water-in-oil-in-water (w/o/w) characteristic to accommodate such API. This method involves preparing a polymeric solution in an immiscible organic solvent alongside an aqueous deionised water phase, with the API introduced into either phase depending on its solubility. Thereafter, two sequential sonication steps are applied to yield CSTHLPLHN-based LPN, with the first generating a w/o nanoemulsion and the second facilitating its dispersion in deionised water. The resulting w/o/w emulsion formulation process concludes with magnetic stirring for solvent evaporation, followed by centrifugal purification.

#### Non-conventional

Beyond conventional approaches, recent developments have introduced alternative one-step techniques within the context of dermatological applications. While the original nomenclature used by respective authors is retained, as highlighted earlier in this section, this review proactively consolidate these methods under the broader category of one-step approaches, characterised by the absence of prior PBN or LBN preparation before direct introduction into their respective counterpart lipid or polymeric phases. These include techniques such as ethanolic injection, self-assembly, ionic gelation, hot melt emulsification, and film rehydration, each distinguished by its sequence of component integration, processing conditions, and the specific type of LPN produced.

Among the various one-step strategies identified, the ethanolic injection technique has been increasingly investigated for its applicability in dermatological LPN development. A notable deviation of this method from the previously discussed traditional approaches lies in using organic solvent to prepare the lipid phase but not the polymeric phase. Fereig et al. [71] formulated polymer-caged nanobin by injecting an ethanolic solution containing lipid, surfactant, and API into an aqueous polymeric solution under continuous magnetic stirring at room temperature. The resulting dispersion was further stirred for 30 min to facilitate nanoparticle formation. Another closely related formulation strategy is the self-assembly method, which follows a similar principle but differs in the sequence of component integration. This approach introduces the dissolved surfactant and API mixture in the aqueous polymeric phase before adding a lipid ethanolic solution [72, 73]. The resulting mixture is subjected to magnetic stirring at ambient temperature and subsequently treated by ultrasonication to yield PCLSHN.

Ionic gelation is another method reported for preparing LPN to treat dermatological conditions. Using this approach, Khan et al. [39]. first dissolved lecithin and the API in ethanol and sonicated the mixture at 50 °C for one hour. Meanwhile, the aqueous polymeric phase was prepared by dissolving chitosan and Tween 80 in 1% acetic acid. The lipid phase was then added to the aqueous phase under continuous stirring, and the resulting dispersion was centrifuged to harvest CST-PLP-HN. While retaining its original nomenclature, the overall approach described by Khan et al. Khan et al. [39] closely resembles the self-assembly methods discussed in previous paragraph, in our opinion. According to Hu et al. [74], the self-assembly of natural polymers relies predominantly on non-covalent interactions, including hydrogen bonding, hydrophobic/hydrophilic interactions, electrostatic forces, and π–π stacking. This definition aligns with previous studies reviewed under the self-assembly category, where LPN were formed through interactions between cationic chitosan polymer and anionic lipids or between cationic lipids and anionic HCP without a crosslinker [40, 61, 73]. On the contrary, the ionic gelation technique requires electrostatic interactions between polymeric ions and a crosslinking agent [75]. For example, the chitosan cationic amine groups can crosslink with the polyanionic sodium tripolyphosphate (TPP) under stirring conditions to form hydrogel nanoparticles [76, 77]. In line with this definition, another LPN formulation relevant to dermatological treatment, reported by Abosabaa et al. [78], first prepared the lipid phase by adding lipids to a TPP-containing crosslinker solution along with API in a 10% ethanolic solution. The lipid phase was subsequently added dropwise into an aqueous chitosan solution under magnetic stirring to formulate polymer-caged nanobins intended for treating cellulite-related dermatological conditions.

Unlike self-assembled or ionically gelated LPN, solid and monolithic LPN represent structurally distinct formats that necessitate alternative fabrication strategies. These systems exhibit procedural parallels to the established methods employed in the preparation of solid lipid nanoparticles (SLN) and nanostructured lipid carriers (NLC) [79]. For solid LPN, several naming conventions have been reported in the literature, including hot homogenisation, one-step hot melt emulsification followed by ultrasonication, and hot emulsion high-shear homogenisation with sonication [80–82]. For consistency, this review refers to these methodological variants collectively as hot melt emulsification. (Table 1) These methods share similar steps that involve melting the solid lipid phase and heating the aqueous polymeric phase to the same temperature before mixing. The resulting emulsion is then subjected to ultrasonication, with high-shear homogenisation occasionally incorporated to enhance dispersion. Finally, the mixture is cooled to allow lipid recrystallisation and form solid LPN. In contrast, monolithic LPN were formulated using a co-dissolution approach, whereby solid lipid, liquid lipid, polymer, and API were dissolved in chloroform and dried to produce a uniform hybrid matrix film [44, 45, 83]. This dried film was subsequently re-dispersed in a heated aqueous surfactant solution and subjected to ultrasonication. The resulting dispersion was rapidly cooled in an ice bath and centrifuged to recover the final LPN. Importantly, Pukale et al. [83] demonstrated the potential for scalability of this method by successfully transitioning their 3 mL sonication-based batch to a 50 mL batch using high-shear and high-pressure homogenisation. Although the authors did not assign an official name to their method, similar procedures have been referred to as the film rehydration technique in other studies developing LPN for cancer treatment [84]. These emerging methods challenge ambiguous classification boundaries and underscore the need for a more standardised and refined framework that captures the diversity of LPN fabrication strategies.

## Dermatological applications

### Skin permeation and dermal drug delivery

By and large, the skin or integumentary system has long been understood to be the largest organ in the human body, encompassing a surface area of 2 m^2^ on average [85, 86]. Serving a multitude of key physiological roles, the skin is known to act as the body’s barrier, safeguarding against harmful external factors including but not limited to foreign pathogens, ultraviolet radiation, and toxic environmental contaminants [85, 86]. Aside from this the skin has an essential role in immunological systems and maintaining homeostasis in the body, regulating numerous vital processes [85, 86]. Over the years, the skin has become a highly attractive target for drug delivery due to several benefits over the oral route such as by-passing the first-pass metabolism and convenience in dosage administration [87]. Although this is mostly used as a case for transdermal delivery of drugs to the systemic circulation, this is also true for topical dermal delivery not intended for systemic absorption [87].

Depending on the desired outcome, active pharmaceutical ingredients (API) may be required to remain on skin surfaces or penetrate deeper layers. Effective dermatological API delivery for the latter presents more significant challenges due to the skin’s complex barrier properties. The skin consists of multiple heterogeneous layers, including the epidermis, dermis and subcutaneous tissue, along with appendages such as hair follicles, sweat glands, and sebaceous glands. Among these structural components, the outermost layer of the epidermis, the stratum corneum (SC or horny layer) serves as the principal barrier to permeation and is understood to be the rate-limiting step in transdermal drug diffusion [88, 89]. The epidermis is primarily composed of keratinocytes that undergo a process of keratinisation, transitioning from the basal layer (stratum basale) through the stratum spinosum and stratum granulosum before reaching the SC [90]. The SC, approximately 10 µm thick, carries a net negative surface charge and comprises three primary components: (1) corneocytes, which are terminally differentiated keratinocytes enriched with natural moisturising factors and encased in a lipid envelope; (2) corneodesmosomes, proteinaceous structures that function as rivets to interconnect the ‘brick-liked’ corneocytes; and (3) an extracellular lipid matrix, which serves as the ‘mortar’ in the characteristic brick-and-mortar arrangement of the SC [89, 91].

**Fig. 4:**
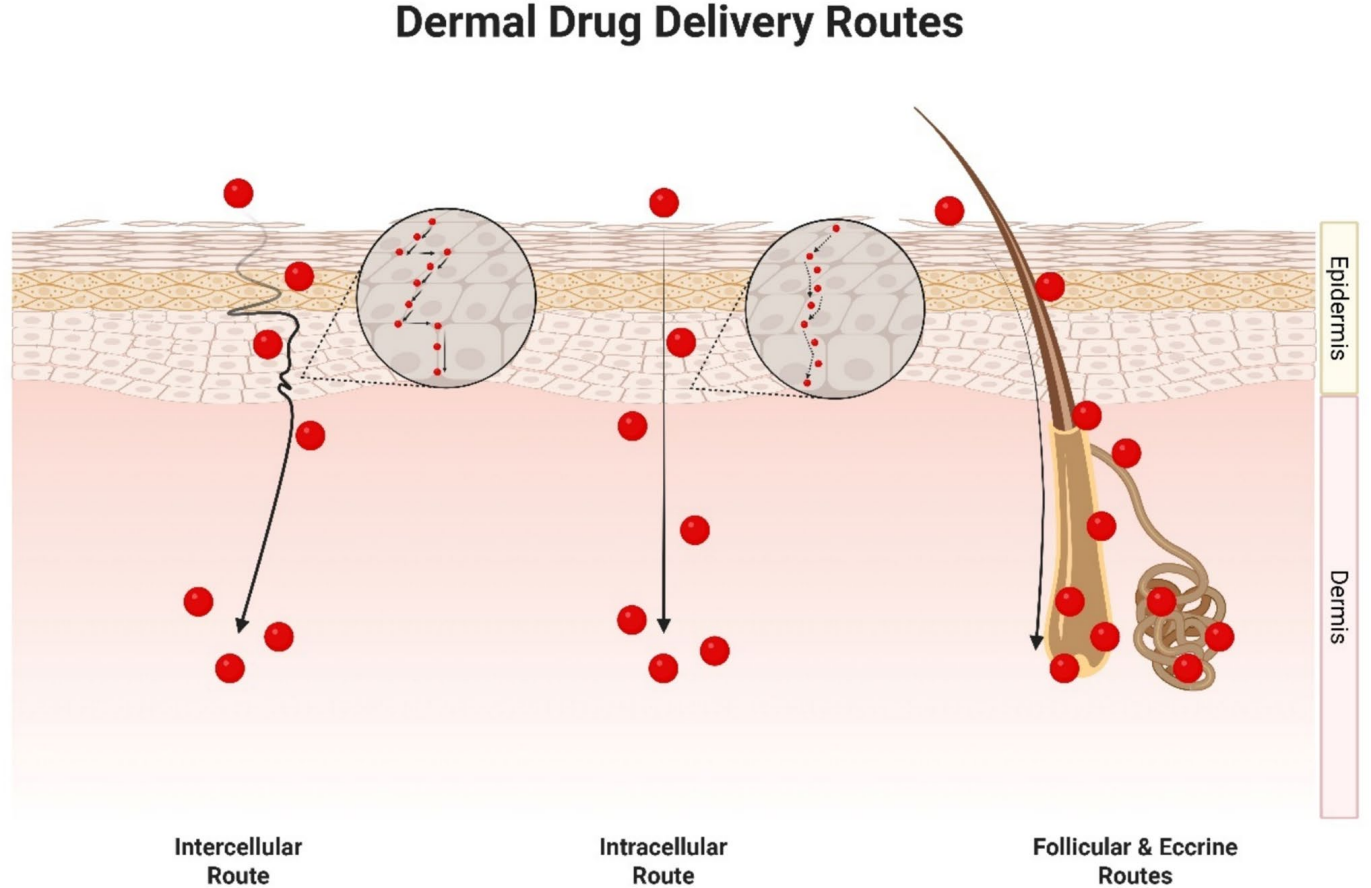
Routes of drug delivery through the skin

With the skin’s formidable barrier properties in mind, nanoparticle systems, including LPN, have gained traction as versatile carriers for transdermal drug delivery. Regardless of whether a substance is a small molecule, large macromolecule, or biological peptide, its transport across the skin is fundamentally limited to a few physiological routes. As illustrated in Fig. 4, three primary pathways have been identified: (1) the intercellular (paracellular) route, (2) the intracellular (transcellular) route, and (3) the follicular and eccrine appendageal routes [87, 92, 93]. LPN, like all nanoparticle systems, are no exception to these rules, and their ability to effectively deliver drugs depends largely on how they interact with these pathways.

Generally, the consensus on intercellular delivery across the skin typically agrees that it is mainly governed by lipophilic moieties [87, 92, 93]. This is largely due to our growing understanding of the structural aspects of the SC layer [87, 92, 93]. From the perspective of any small molecular compounds, for any substance to be transported through the intercellular route, they must traverse a hydrophobic ocean of lipids composed of fatty acids with varying chain lengths, cholesterol, and ceramides [87, 92, 93]. A recently announced unified model of the SC nanostructure, supported by large amounts of research has recently been proposed [94]. Hardly any aqueous regions are available within this sea of lipids, and if present, are used for hydrogen bonding of polar head groups of lipids in the SC to support the formation of a tight network [93].

Unlike intercellular delivery, the intracellular route is less understood with relatively scarce amounts of empirical evidence studying the diffusion through the skin using this route [93]. On top of the complex microenvironment of the SC layer, studying intracellular delivery within this region requires consideration of countless other factors surrounding cell biology and structure, tight junction protein behaviour, and cell signalling interactions to name a few [93]. However, experimental data on highly hydrophilic molecules such as urea, amino acids, and small peptides, does support the idea that the intracellular route is typically a path taken by hydrophilic moieties which are unable to gain access to the intercellular path [93].

Lastly, the follicular and eccrine routes of dermal drug delivery are niche areas useful for specific targeting in certain clinical conditions such as alopecia but generally have low extents of drug delivery to the skin overall [87]. Regions closer to hair follicle openings are known to possess thinner sections of the SC layer [87]. However, often the active presence of a sebaceous plug serves as a limiting factor, impeding the transport of substances through this route [87]. Certain strategies have been taken to improve transport of materials through this route such as iontophoresis due to the lower electrical resistance surrounding the cutaneous appendages relative to the rest of the SC layer [87].

As highly adaptable nanoparticle system, LPN present a unique advantage in dermal drug delivery through the tunability of their surface physicochemical properties. Additionally, the lipophilic nature of their lipid excipients enhances their compatibility with the intercellular lipid matrix of the stratum corneum, facilitating passage through this dominant barrier. These modifiable features allow LPN to overcome limitations posed by the intrinsic properties of various therapeutic cargos, regardless of whether they are hydrophilic or hydrophobic. This flexibility enables LPN to target and deliver drugs more effectively through one or more of the skin’s established permeation routes.

### Safety and biocompatibility

Preceding therapeutic efficacy, the safety and biocompatibility of pharmaceutical formulations is paramount when developing a new treatment strategy. For dermatological conditions, considerations for skin irritation and tolerability represent unique aspects aside from the general toxicity profiles [95].

From a practical perspective, both lipid and polymer components of LPN are typically selected from materials with established safety profiles, such as phospholipids and PLGA or chitosan, minimizing the risk of adverse skin reactions. Lipids, which closely resemble components of the skin’s natural barrier, help to reduce irritation and immunogenicity, while the biodegradable polymers degrade into non-toxic byproducts, further supporting their safe application on the skin [95].

Studies evaluating the dermal application of LPN have generally reported low in vitro cytotoxicity toward skin cell lines, such as keratinocytes and fibroblasts [40, 96]. For instance, Rahman et al. found their developed multilamellar collagen-lipid hybrid vesicles (MCLV) incorporated with all-trans-retinol (ATRL) displayed acceptable biocompatibility in human dermal fibroblast (HDF) cells [40]. In fact, the MCLV incorporating ATRL outperformed both free ATRL and large unilamellar vesicles (LUV) incorporating ATRL in terms of safety [40]. This interesting finding was likely due to the reduction in particle density and total surface area in contact with cell membranes granted by the MCLV formulation [40]. Notably, blank LUV displaying significant toxicity is likely due to the presence of cationic DOTAP lipids which are known to disrupt cellular membranes through their strong electrostatic interactions [40]. However, the incorporation of collagen along with the modification of the particle architecture to form MCLV reduced the toxicity of the formulation significantly [40]. A separate study using fusidic acid-loaded nanoengineered lipid-polymer hybrid nanoparticles (FA-LPHN) similarly found acceptable safety profiles when tested in vitro on an immortalised human keratinocyte cell line (HaCaT) [96]. A limitation of this study is the lack of comparison between the lipid component and the polymer component of the formulation with the final LPN formulation itself. Unfortunately, this is a common trend with LPN studies in dermatological conditions.

Meanwhile, in vivo safety and toxicity studies have also shown excellent tolerability of LPN towards the skin [71, 97]. Alsaidin et al. demonstrated using in a rabbit model that the developed hydrocortisone-loaded LPN displayed acceptable skin tolerability which was determined through histopathological analysis [97]. Authors of the study reported the absence of any neutrophil invasion along with greater thickening of the epidermis layer in rabbits treated with the optimised formulation [97]. The occlusive nature of the formulation attributed to the phospholipid component of the LPN was the explanation for this observation by the authors [97]. In a separate study, mice (HRS/J, Jackson Laboratories USA) were used to determine the safety of LPN carrying TNF-α siRNA coupled with a photosensitiser (TPPS2a) [71]. Histopathological analysis had shown the skin of mice treated with the developed formulation were most similar to those of normal skin (control, healthy), with an absence of large increases in epidermal thickening and leukocyte infiltration [71]. Authors of this study had remarked that despite the general safety of LPN formulations, researchers should be cautious as higher concentrations of cationic polymers such as poly(allylamine hydrochloride) have evidently demonstrated toxicity in other literature [71, 98–101].

### Clinical and cosmetic applications

**Fig. 5:**
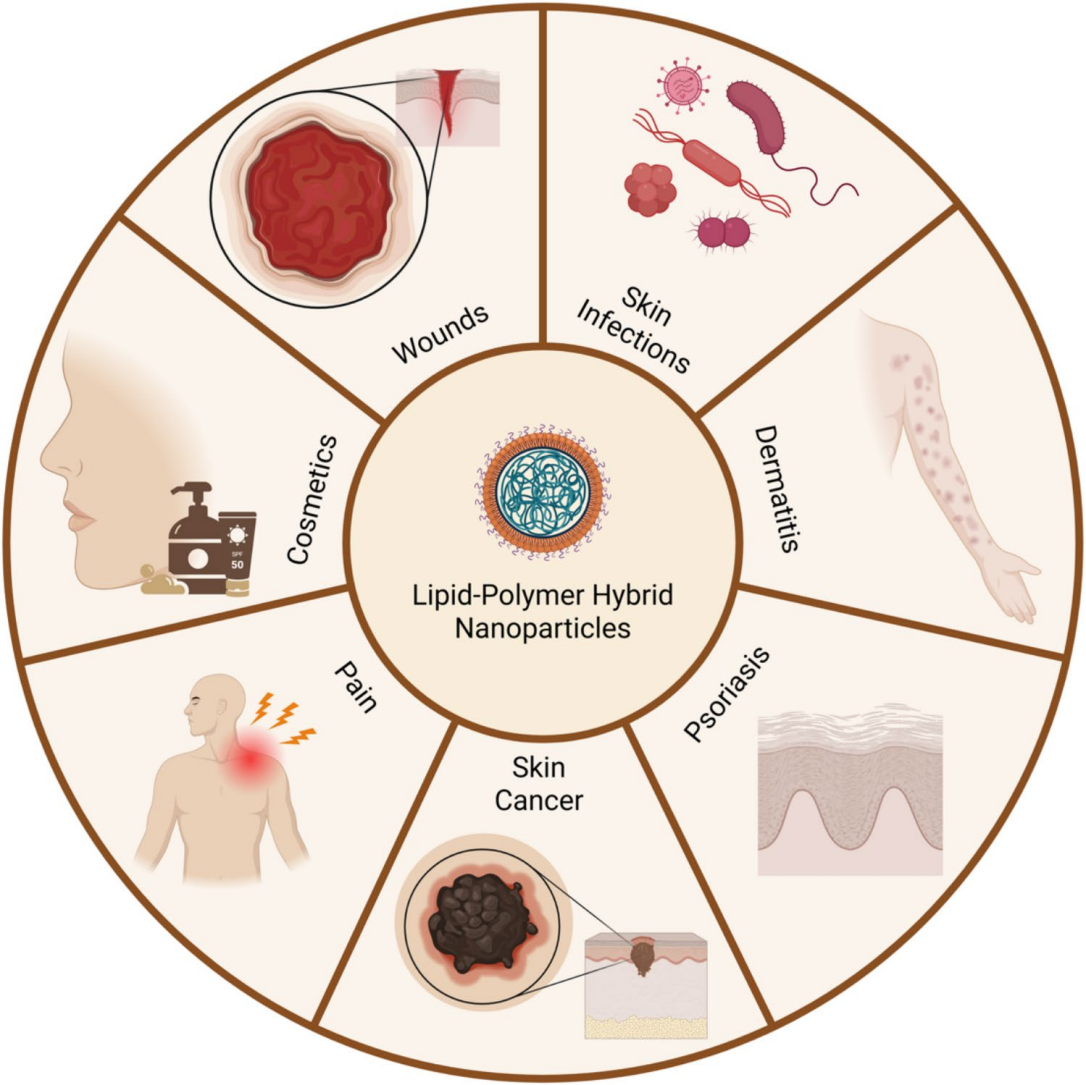
Clinical and cosmetic applications of LPN in dermatology and skincare

Building upon the mechanistic insights into nanoparticle transport across the skin barrier discussed in the preceding section, this part of the review examines the practical implementation of LPN in dermatological and cosmetic applications. The adaptability of LPN systems to enhance skin permeability and drug release as well as modulate drug release has led to their investigation across a wide range of dermatological conditions. Table 3 presents a consolidated summary of recent LPN-based strategies tailored for skin-related indications, detailing formulation components, target applications, and observed outcomes. The subsequent subsections explore these applications in greater depth, focusing on therapeutic areas such as wound healing, infections, dermatitis, psoriasis, skin cancer, pain management, and cosmetics. (Fig. 5).

**Table 3:** Recent advances in Lipid-Polymer Nanoparticles (LPN) for dermatological applications

Types of LPN	Methods of Formulation	Main Compositions of LPN	Characteristics of The Optimised LPN	Storage Stability of The Optimised LPN	Final Forms of Formulation	Dermatological Applications	References
Core shell-type hollow lipid-polymer-lipid hybrid nanoparticle*	Double-emulsification-solvent-evaporation	API(s): Hisperidine Lipid(s): Soya lecithin Surfactant(s): Polyvinyl alcohol Lipid-PEG: N/A Polymer(s): Chitosan Conjugate(s): N/A	PS: 91.43 nm PdI: 0.056 ZP: 15.6 mV EE%: 92.8 LC%: N/A	No significant changes were observed in ZP and EE% when stored at 4 °C and 25 °C for three months, with only a negligible increase in PS	Suspension	Wound	[102]
Polymer core lipid-shell hybrid nanoparticle*	Self-assembly	API(s): Acyclovir Lipid(s): Glyceryl monooleate Surfactant(s): Span 80 Lipid-PEG: D-α-tocopheryl polyethylene glycol succinate Polymer(s): Medium molecular weight chitosan Conjugate(s): N/A	PS: 177.50 ± 1.41 nm PdI: 0.28 ± 0.02 ZP: − 10.70 ± 0.85 mV EE%: 77.20 ± 2.40% LC%: N/A	After three months of storage at 25 ± 2 °C and 60 ± 5% RH, PS and PdI increased significantly, whereas ZP and EE% remained stable. Conversely, at 5 ± 3 °C, all measured properties showed no significant changes	Suspension	Viral infection	[73]
Polymer core lipid-shell hybrid nanoparticle*	Self-assembly	API(s): Ceftriaxone sodium Lipid(s): Glycerol monostearate Surfactant(s): Tween 80 Lipid-PEG: N/A Polymer(s): Medium molecular weight chitosan Conjugate(s): N/A	PS: 284 ± 1 nm PdI: 0.20 ± 0.02 ZP: 15 ± 2 mV EE%: 79 ± 0.7 LC%: 19.17 ± 0.7	N/A	Dry powder	Bacterial infection	[72]
Core shell-type polymer-lipid-polymer hybrid nanoparticle*	Ionic gelation	API(s): Ketoconazole Lipid(s): Lecithin Surfactant(s): Tween 80 Lipid-PEG: N/A Polymer(s): Chitosan Conjugate(s): N/A	PS: 173 nm PdI: 0.177 ZP: positive charge (not specified) EE%: < 70% (not specify) LC%: N/A	N/A	Hydrogel	Fungal infection	[39]
Polymer core lipid-shell hybrid nanoparticle	Nanoprecipitation	API(s): Hydrocortisone Lipid(s): Phospholipon 90 G Surfactant(s): Tween 80 Lipid-PEG: N/A Polymer(s): Polycaprolactone Conjugate(s): N/A	PS: 249.7 nm PdI: N/A ZP: –27.3 mV EE%: 82.7 LC%: N/A	No significant changes were observed in PS, ZP and EE% when stored at 4 ± 0.5° and 25 ± 1 °C for one months	Suspension	Atopic dermatitis	[97]
Polymer-caged nanobin	Ethanolic injection	API(s): Tacrolimus Lipid(s): Lecithin Surfactant(s): Tween 80 Lipid-PEG: N/A Polymer(s): Medium molecular weight chitosan Conjugate(s): N/A	PS: 118.70 ± 13.3 nm PdI: 0.43 ± 0.13 ZP: 16.20 ± 2.40 mV EE%: 66.72 ± 1.80 LC%: 18.20 ± 0.50	After 3 months of storage at 4 °C, the formulation exhibited significant instability. PS and PdI increased more than twofold, reaching 273.06 ± 35 nm and 0.98 ± 0.001, respectively. ZP dropped to 10.5 ± 2.1 mV, and EE% decreased by approximately 15%	Suspension	Psoriasis	[71]
Polymer core lipid-shell hybrid nanoparticle	Single emulsification-solvent-evaporation	API(s): Curcumin Lipid(s): Stearic acid Surfactant(s): Tween 80 Lipid-PEG: N/A Polymer(s): Ethyl cellulose Conjugate(s): N/A	PS: 200.9 nm PdI: 0.342 ZP: −28.3 mV EE%: 87.40 ± 0.99 LC%: 4.57 ± 0.04	After 3 months of storage under refrigerated conditions (4 ± 2 °C, 75 ± 5% RH) and normal conditions (25 ± 2 °C, 75 ± 5% RH), the following observations were made: PdI increased slightly to 0.383 and 0.359, respectively, while EE% also increased slightly to 88.76 ± 0.50 and 90.50 ± 1.20. Interestingly, PS decreased to 158.0 nm under refrigerated conditions but increased to 269.2 nm under normal conditions	Hydrogel	Psoriasis	[103]
Polymer core lipid-shell hybrid nanoparticle	Single emulsification-solvent-evaporation	API(s): Methoxsalen and curcumin Lipid(s): Stearic acid Surfactant(s): Tween 80 Lipid-PEG: N/A Polymer(s): Ethyl cellulose Conjugate(s): N/A	PS: 206.8 ± 3.2 nm PdI: 0.174 ZP: − 27.1 ± 6.09 mV EE%: 83.85 ± 0.73 (methoxsalen); 84.90 ± 0.68 (curcumin) LC%: 2.87 ± 0.82 (methoxsalen); 2.61 ± 0.35 (curcumin)	Over a 3-month period, the formulation demonstrated varying stability at the fridge (4 °C, 75 ± 5% RH) and room temperature (25 °C, 75 ± 5% RH). At fridge temperature, particle size increased slightly to 224.3 ± 1.7 nm, PdI rose significantly to 0.254 ± 0.012, and EE% decreased to 79.98 ± 0.46. At room temperature, the formulation showed marked instability with particle size increasing to 309.3 ± 6.2 nm, EE% dropping sharply to 58.01 ± 0.82, and PdI rising significantly to 0.257 ± 0.010	Hydrogel	Psoriasis	[104]
Monolithic lipid-polymer hybrid nanoparticle	Film rehydration**[84]	API(s): Vitamin D3 Lipid(s): Precirol® ATO 5 and linoleic acid Surfactant(s): Tween 80 Lipid-PEG: N/A Polymer(s): di-block copolymer of DL-lactide and Methoxy poly(ethylene glycol) Conjugate(s): N/A	PS: 123.1 ± 6.16 nm PdI: 0.234 ± 0.03 ZP: − 4.33 ± 0.85 mV EE%: 76.80 ± 1.36 LC%: N/A	N/A	Hydrogel	Psoriasis	[44]
Monolithic lipid-polymer hybrid nanoparticle	Film rehydration**[84]	API(s): Coenzyme Q10 Lipid(s): Precirol® ATO 5 and linoleic acid Surfactant(s): Tween 80 Lipid-PEG: N/A Polymer(s): di-block copolymer of DL-lactide and Methoxy poly(ethylene glycol) Conjugate(s): N/A	PS: 121 ± 11.61 nm PdI: 0.252 ± 0.073 ZP: − 20.23 ± 6.67 mV EE%: 78.57 ± 3.88 LC%: N/A	N/A	Hydrogel	Psoriasis	[45]
Polymer core lipid-shell hybrid nanoparticle	Self-assembly	API(s): Gallic acid Lipid(s): Soy-lecithin Surfactant(s): N/A Lipid-PEG: N/A Polymer(s): Low molecular weight chitosan Conjugate(s): N/A	PS: 170.5 ± 0.087 nm PdI: 0.19 ± 0.0015 ZP: − 27.97 mV EE%: 63.57 ± 0.001 LC%: N/A	N/A	Hydrogel	Psoriasis	[61]
Polymer core lipid-shell hybrid nanoparticle	Single emulsification-solvent-evaporation	API(s): Rapamycin Lipid(s): Phospholipon 90G Surfactant(s): Tween 80 Lipid-PEG: D-α-tocopheryl polyethylene glycol succinate Polymer(s): Poly(lactic-co-glycolic acid) copolymer of DL-lactide and Glycolide in a 50:50 molar ratio (MW-7,000–17,000 da) and polyvinyl alcohol Conjugate(s): N/A	PS: ≈ 277.6 nm PdI: 0.21 ZP: ≈ − 17.5 mV EE%: ≈ 83 LC%: N/A	N/A	Hydrogel	Psoriasis	[105]
Solid lipid-polymer hybrid nanoparticle*	Hot-melt emulsification	API(s): Photosensitiser (TPPS2a) Lipid(s): Compritol® 888 ATO Surfactant(s): Poloxamer 188 Lipid-PEG: N/A Polymer(s): poly(allylamine hydrochloride) Conjugate(s): Tumor necrosis factor α siRNA	PS: 253.7 ± 5.8 nm PdI: 0.340 ± 0.015 ZP: 26.4 ± 2.7 mV EE%: 99.51 ± 0.35 LC%: N/A	N/A	Suspension	Psoriasis	[80]
Solid lipid-polymer hybrid nanoparticle	Hot-melt emulsification	API(s): Solamargine and solasonine Lipid(s): Crodamol SS™ Surfactant(s): N/A Lipid-PEG: N/A Polymer(s): Cationic polymer P1 Conjugate(s): N/A	PS: 195.6 ± 3.5 nm PdI: 0.155 ± 0.01 ZP: 37.2 ± 0.5 mV EE%: 99.99 ± 0.02 (solamargine); 99.99 ± 0.02 (solasonine) LC%: N/A	After six months of storage at low temperatures (−20 °C and 4 °C), PdI remained below 0.2, and the ZP ranged between 35 and 40 mV, with no significant variations observed under either condition. The mean PS was approximately 200 nm at both temperatures, although the change in PS was statistically significant when stored at −20 °C	Suspension	Melanoma	[81]
Lipid core polymeric nanocapsules/Lipomer*	Single emulsification-solvent-evaporation	API(s): Dacarbazine Lipid(s): Maisine 35–1 Surfactant(s): Tween 80 Lipid-PEG: N/A Polymer(s): Poloxamer 188 Conjugate(s): N/A	PS: 202.7 nm PdI: N/A ZP: − 24.89 mV EE%: 70.29 LC%: N/A	N/A	Hydrogel	Melanoma	[34]
Polymer core lipid-shell hybrid nanoparticle	Nanoprecipitation	API(s): Lidocaine hydrochloride Lipid(s): Lecithin Surfactant(s): N/A Lipid-PEG: 1,2-distearoyl-sn-glycero-3-phosphoethanolamine-N-[methoxy(polyethylene glycol)−2000] (DSPE-PEG2000) Polymer(s): Poly(lactic-co-glycolic acid) Conjugate(s): N/A	PS: ∼175 ± 3 nm PdI: 0.15 ± 0.02 ZP: −35 ± 4 mV EE%: 90 ± 5 LC%: 91 ± 6	N/A	Suspension	Anesthetics and analgesic	[106]
Polymer-caged nanobin*	Ionic gelation	API(s): Caffeine, catechin (C), epicatechin (EC) and epigallocatechingallate (EGCG) Lipid(s): Lecithin Surfactant(s): N/A Lipid-PEG: N/A Polymer(s): Medium molecular weight chitosan Conjugate(s): N/A	PS: 292.6 ± 8.98 nm PdI: 0.253 ± 0.02 ZP: 41.03 ± 0.503 mV EE%: 68.4 ± 1.88 LC%:	N/A	Suspension	Anti-cellulite (orange-peel)	[78]
Polymer-caged nanobin*	Self-assembly**[40, 61]	API(s): Octyl methoxycinnamate Lipid(s): Phosal® 53 MCT Surfactant(s): Tween 80 Lipid-PEG: N/A Polymer(s): Low molecular weight chitosan Conjugate(s): N/A	PS: Three size populations of 75.7 ± 5.9 nm, 515.4 ± 21.8 and 4726.0 ± 291.3 PdI: 0.49 ± 0.01 ZP: 10.6 ± 0.4 mV EE%: 97.1 ± 0.3 LC%: N/A	Minimal changes were observed in the size distribution profile of the three size populations after 90 days of storage	Hydrogel	Sunscreen	[62]
Lipid core polymeric nanocapsules/Lipomer	Single emulsification-solvent-evaporation	API(s): Dexamethasone Lipid(s): Medium chain triglycerides Surfactant(s): Span 60 and Tween 80 Lipid-PEG: N/A Polymer(s): Ethyl cellulose Conjugate(s): N/A	PS: 374.33 ± 7.60 nm PdI: 0.229 ± 0.011 ZP: 34.7 ± 0.4 mV EE%: 98.87 ± 0.01 LC%: N/A	N/A	Hydrogel	Alopecia	[35, 36]^‡^
Polymer core lipid-shell hybrid nanoparticle	Two-step technique	API(s): miR-218 Lipid(s): Lecithin and cholesterol Surfactant(s): N/A Lipid-PEG: Distearoyl phosphoethanolamine-polyethylene glycol (DSPE-PEG) Polymer(s): Polyvinylamine, Xelorex™ RS 1100 Conjugate(s): N/A	PS: 141 ± 14 nm PdI: 0.16 ± 0.04 ZP: 5.34 ± 1.06 mV EE%: N/A LC%: N/A	N/A	Microneedle patch	Alopecia	[107]
Multilamellar nanovesicle	Self-assembly	API(s): All-trans-retinol Lipid(s): 1,2-dioleoyl-3-trimethylammoniumpropane (DOTAP) Surfactant(s): N/A Lipid-PEG: N/A Polymer(s): Hydrolyzed collagen peptides Conjugate(s): N/A	PS: 142.9 ± 5.4 nm PdI: N/A ZP: 28 mV^†^ EE%: 97.5 LC%: N/A	Stability assessments over 90 days at 4, 25, and 37 °C revealed that LPN effectively prevented API degradation at 4 and 25 °C. However, at 37 °C, the API degraded significantly, with less than 30% remaining after 90 days of storage	Suspension	General cosmetic	[40]
Solid lipid–polymer hybrid nanoparticle	Hot-melt emulsification	API(s): Silencer™ Negative Control siRNA (siRNA) Lipid(s): Compritol® 888 ATO Surfactant(s): Poloxamer 188 Lipid-PEG: N/A Polymer(s): Branched polyethylenimine (25 kDa) Conjugate(s): N/A	PS: 175.15 ± 17.71 nm PdI: 0.29 ± 0.12 ZP: 27.18 ± 1.55 mV EE%: N/A LC%: N/A	Stability assessments at 4 °C and 30 °C with 75% relative humidity over 90 days showed slight changes in PS at both temperatures. ZP remained stable at 4 °C but became more positively charged at 30 °C	Suspension	N/A	[82]

Superscripts:

* Information is based on general descriptions, illustration figures, beliefs, or discussions by the authors. It was not verified using validated methods, and the authors do not give a clear statement on the type of LPN they formulated

** The method name used here is referenced from other sources, as the original study did not specify an official name for the formulation approach they employed

† Values were estimated from graphs or figures as no specific results or labels were explicitly reported

‡The summarised results presented here represent the final formulation, derived from a series of sequential studies conducted by the same research group

*API* Active pharmaceutical ingredient, *EE%* Encapsulation efficacy, *LC%* Loading capacity, *N/A* Not available, *PEG* Polyethylene glycol, *PdI* Polydispersity index, *PS* Particle size, *RH *Relative humidity, *ZP *Zeta potential

#### Wound

Chronic wounds represent a persistent global health challenge, placing a considerable burden on healthcare systems and incurring high socioeconomic costs [108]. In response to these clinical needs, Romić et al. [109] explored the therapeutic potential of lipid–polymer hybrid systems by leveraging the intrinsic wound-healing properties of NLC together with the regenerative, haemostatic, and antimicrobial properties of chitosan [110, 111]. They developed a hybrid delivery system by integrating Compritol® 888 ATO and Miglyol® 812 N into a chitosan matrix loaded with melatonin. Although spray-drying resulted in microspheres (< 4 µm) formulation rather than nanoparticles, it demonstrated notable biological healing efficacy. Their in vitro scratch assays performed using the human diploid fibroblast strain MJ90hTERT revealed significantly enhanced wound closure with the lipid-enriched chitosan microspheres compared to both untreated controls and chitosan-only microspheres.

The study presents encouraging in vitro evidence and supports the strategic integration of lipid and polymer components within a unified micron-size delivery platform for improved wound management. However, the lack of in vivo evaluation limits its translational potential. Given that the LPN in this study was prepared in the micron size range, its therapeutic suitability would probably lie within the treatment of superficial wounds, where localised retention and sustained drug release are desirable. Microparticles tend to remain at the application site, offering a depot effect that maintains therapeutic levels over an extended period while minimising systemic exposure [112, 113]. In contrast, nanoparticles possess enhanced tissue penetration capabilities and may be more appropriate for chronic or infected wounds that require delivery into deeper dermal layers [113, 114]. However, this advantage comes with an increased risk of systemic absorption, which must be carefully optimised. Therefore, the absence of in vivo data in this study limits the ability to fully assess the clinical relevance of the microscale LPN system, particularly in relation to the different types of wounds that are complex and dynamic in nature.

Taking a comparable direction, Jangde et al. [102] developed a LPN system for delivering hesperidin, a bioflavonoid with recognised wound-healing properties. Although the authors acknowledged the contribution of chitosan to wound repair through its ability to accelerate cell proliferation, facilitate tissue reorganisation, and inhibit microbial growth, the study did not include in vitro or in vivo wound healing models. Instead, the primary objective was to address hesperidin’s poor aqueous solubility and limited topical bioavailability through a novel formulation. The authors selected LPN based on their superior solubility and drug-dispersing capabilities compared to conventional nanoformulations. Through a design of experiments approach, they optimised the LPN formulation to achieve a particle size of 91.43 nm, a polydispersity index (PdI) of 0.056, a zeta potential (ZP) of 15.6 mV, and an encapsulation efficiency (EE%) of 92.8%. In vitro release studies confirmed a sustained release profile, with approximately 80% of the drug released over 24 h.

#### Infections

LPN have gained considerable attention in the topical treatment of dermatological infections of bacterial, viral, and fungal origin. One notable application is against *Staphylococcus Aureus* (SA) and Methicillin-Resistant *Staphylococcus Aureus* (MRSA), which are commonly implicated in burn wound infections and represent a significant cause of morbidity in thermally injured patients [96, 115]. Eschar formation in burn wounds impedes drug permeation and immune responses, while the avascular nature of these wounds further limits systemic antibiotic bioavailability, necessitating more effective localised therapeutic strategies [115]. In response, fusidic acid (FA)-loaded LPN incorporating chitosan was developed to leverage wound healing and permeation-enhancing properties of the carrier’s system [96]. The optimised formulation demonstrated sustained drug release and was non-cytotoxic to HaCaT keratinocyte cells. Remarkably, it achieved approximately fivefold, fourfold, and 3.6-fold reductions in the minimum inhibitory concentrations (MIC) against *MRSA 33591*, *MSSA 25921*, and *SA 22359*, respectively [96]. Subsequent incorporation into a carbopol gel further enhanced its skin permeation (76.53 ± 1.55%) and drug retention (56.41 ± 4.67%) compared to conventional formulations [115]. In vivo evaluation in a murine model of MRSA-infected burn wounds revealed a marked reduction in bacterial burden (5.22 log CFU/mL by day 3) and significantly improved wound contraction (68.70 ± 3.65% by day 5) following topical application of the FA-LPN gel, outperforming both untreated controls and standard FA cream [115]. These findings underscore the importance of recognising not only the drug delivery advantages of LPN but also the pharmacological contributions of their excipients. In particular, incorporating chitosan has been shown to actively promote wound healing in burn-related infections. Such effects should be deliberately considered during formulation design to ensure that the selected materials not only facilitate delivery but also directly support the therapeutic objectives. While this study did not investigate whether the lipid components contributed to the therapeutic effects, as demonstrated with wound-healing NLC in the preceding section, it nonetheless highlights the value of formulating LPN with bioactive materials that align with the clinical context. Collectively, these findings support LPN as a promising and rational delivery strategy to overcome the limitations of conventional therapies and improve the management of resistant skin infections.

In another study, Dave et al. [116] formulated norfloxacin-loaded LPN targeting general infection associated with *Staphylococcus aureus* and *Pseudomonas aeruginosa*. Unlike the prior work that focused on burn-associated SA and MRSA infections, this study primarily emphasised the controlled-release properties of LPN. The optimised formulation exhibited a biphasic drug release profile, with approximately 83% of norfloxacin released steadily within the first 8 h, followed by a sustained release of the remaining 7% over the next 16 h. Antimicrobial assays demonstrated enhanced activity of the LPN formulation, achieving approximately 90% zone of inhibition efficacy relative to the free drug against both bacterial strains. Drawing from this work, Abadi et al. [72] encapsulated the hydrophilic antibiotic ceftriaxone into LPN for cellulitis treatment, using *Escherichia coli* and *Enterococcus faecium* as representative Gram-negative and Gram-positive bacteria. The study highlighted the influence of the lipid-to-polymer (L/P) ratio on drug release kinetics and nanoparticle crystallinity, where an optimised L/P ratio (0.5) facilitated more controlled drug release and improved antibacterial efficacy. In particular, the MIC of ceftriaxone against *E. coli* was reduced from 2 μg/mL (free drug) to 1 μg/mL in the optimised LPN, suggesting a release-governed enhancement in therapeutic effect. Moreover, the kinetic death profile of *E. coli* showed a continuous reduction in bacterial count up to 10 h of the final study time point. On the contrary, other L/P ratios exhibited a plateau after 6 h, suggesting that bacterial killing persisted longer with the optimised controlled released LPN. When tested against *E. faecium*, the optimised LPN exhibited approximately 50% lower bacterial resistance than conventional antibiotics such as cefixime, cefalexin, ceftriaxone, ceftizoxime, and cefazolin. These findings provide rare yet compelling evidence that sustained drug release offers more than improved patient compliance, demonstrating a direct and measurable impact on clinical treatment efficacy. In this context, the prolonged release notably enhanced antimicrobial performance. This highlights the value of LPN as a delivery platform due to their inherent ability to modulate physicochemical characteristics and precisely tailor drug release behaviour to meet specific therapeutic needs.

In recent years, the potential of LPN systems in enhancing skin permeation for bacterial infection treatments has extended to the topical delivery of acyclovir (ACR) for managing herpes simplex virus (HSV) and varicella-zoster virus (VZV) infections. Recognising the promising results from earlier studies, Abd-Elsalam et al. [73] took a step further by incorporating Span 80 and D-α-tocopheryl polyethylene glycol succinate (TPGS) as surfactants to enhance skin penetration by disrupting the structural integrity of lipids and proteins in the stratum corneum. However, despite achieving a threefold increase in AUC₀_–_₂₄ compared to the ACR suspension, it remains unclear whether therapeutic concentrations were attained at the primary infection site of the basal epidermis. This additional information is crucial, as earlier findings from the 1990 s reported that a topical ACR formulation with tenfold higher epidermal delivery concentration failed to produce the desired therapeutic effect when compared to orally administered ACR [117]. Nevertheless, the modified Draize test and histopathological analysis confirmed the safety profile of the LPN, revealing no signs of inflammation or necrosis following topical application.

Similarly, Khan et al. [39] recently expanded the anti-infection spectrum of LPN by fabricating a ketoconazole-loaded LPN hydrogel formulation intended for the topical treatment of fungal infections. A series of evaluations was conducted to establish the formulation’s safety and therapeutic performance. Dermatological safety was confirmed via skin irritation studies in albino rats, wherein the LPN’s primary dermal irritation index (PDII) remained below 0.5 compared to the formalin-treated control group of more than five, signifying a non-irritant classification for the formulation. On the other hand, the therapeutic efficacy was examined through antifungal susceptibility testing against *Aspergillus niger* using a disc diffusion method. Although the authors did not report precise inhibition zone diameters, they asserted that the LPN hydrogel exhibited markedly greater antifungal activity than the commercially available ketoconazole gel based on visual inspection. Furthermore, ex vivo permeation studies employing excised rat skin demonstrated that approximately 8% of ketoconazole permeated the LPN formulation after 12 h, compared to 3% from the LPN gel and 1% from a simple drug dispersion. Notably, the authors did not explain the comparatively reduced permeation observed with the LPN gel, leaving the mechanistic basis for this observation unaddressed.

#### Dermatitis

Atopic dermatitis is a chronic and relapsing inflammatory skin disorder characterised by intense pruritus, impaired skin barrier function, xerosis, and eczematous inflammation [118]. Effective management requires consistent skin care and pharmacological intervention. Hydrocortisone acetate (HCA), a topical corticosteroid, is frequently used but suffers from poor skin permeability when applied as a raw compound [119]. To enhance its dermal delivery, Alsaidan et al. [97] developed LPN by encapsulating HCA within the biodegradable lipophilic polymer polycaprolactone and coating the polymeric core with Phospholipon 90 G. This phospholipid component mimics skin lipids and facilitates penetration through the stratum corneum.

Their ex vivo studies confirm that this formulation strategy significantly improved HCA’s flux and permeability coefficient compared to a plain HCA solution. Furthermore, histological analysis following topical application revealed increased epidermal thickness with minimal dermatotoxicity, indicating an occlusive effect from the phospholipid-rich LPN. This occlusion likely disrupts the skin’s primary barrier and promotes drug permeation, consistent with the enhanced cutaneous delivery observed in the ex vivo assays. In vivo, the formulation demonstrated potent anti-inflammatory activity in a croton oil-induced rosacea model. These findings support using LPN as an effective strategy to improve the topical bioavailability and therapeutic efficacy of corticosteroids like HCA.

#### Psoriasis

Presenting as a chronic papulosquamous skin disease, the pathogenesis of psoriasis has been determined to primarily involve inflammation of the skin modulated by numerous immunological pathways with underlying genetic predispositions [120, 121]. Currently, a wide range of topical pharmaceutical therapies are available to manage psoriasis, including corticosteroids, vitamin D analogues, retinoids, calcineurin inhibitors, salicylic acid, and coal tar [122]. However, despite the myriad of treatment options available at our disposal, the available choices can only provide symptomatic relief and the occurrence of treatment failure or refractory cases are not uncommon in clinical practice [122, 123].

Throughout the years, numerous studies have investigated the use of LPN to improve drug delivery and ultimately therapeutic outcomes. Two such studies have examined the use of lecithin-chitosan hybrid nanoparticles for this purpose, employing two systems despite having the same primary components [61, 71]. Fereig et al. [71] developed a lecithin-chitosan hybrid nanoparticle comprising a hydrophobic lecithin core and a hydrophilic cationic chitosan shell structure, further stabilised with either Tween 80 or olive oil as a co-solvent to encapsulate tacrolimus, a notoriously hydrophobic compound. Meanwhile, Hazari et al. [61] utilised a hydrophilic chitosan core and a amphiphilic lecithin-coated shell in their design to encapsulate gallic acid, a hydrophilic phytochemical. The differences in the nanoparticle structure between these two studies is evident in the difference in the polarity of the electrical charge of these 2 systems (Table 1), highlighting the versatility of LPN-based nanoparticle systems. Interestingly, both studies demonstrated favourable but slightly different outcomes in dermal penetration [61, 71]. The system with an outer cationic chitosan shell was able to improve the deposition and retention of tacrolimus within the stratum corneum and epidermis, with little to no drug reaching the dermis while being retained up to 75% when tested in an in vivo model [71]. Meanwhile tacrolimus without the formulation permeated the entire skin layer past the dermis with minimal drug retention, 13.8% when tested in vivo [71]. On the other hand, the system with an outer lecithin shell, improved penetration of gallic acid into deeper layers of the skin while gallic acid on its own had limited permeation into the skin [61]. Both studies showed superior anti-psoriatic activity, however, no differences were seen in immunogenic response based on spleen to body weight ratio between the drug alone versus the formulation group [61].

Another two studies by Jaiswal et al. and Jamatia et al. investigated the use of a stearic acid-ethyl cellulose blend of LPN prepared through similar methods to encapsulate curcumin and co-encapsulate methoxsalen and curcumin, respectively [103, 104]. Both studies achieved closely similar nanoparticle properties (Table 1) and observed similar biological behaviours. Although both studies reported permeation and retention of curcumin or curcumin and methoxsalen using an ex vivo pig ear skin model, the lack of a control group for comparison raises ambiguity in interpretation [103, 104]. In terms of anti-psoriatic effects, by using an in vivo imiquimod (IMQ)-induced psoriasis Wistar rat model, both authors demonstrated improved outcomes comparably better than a marketed product (Betamethason Valerate 0.1% w/w cream) through visual inspection, scoring, and histopathological analysis [103, 104]. The authors from these 2 studies attributed their findings to the therapeutic efficacy of their cargo and enhanced drug retention in the skin [103, 104].

Approaching the matter from a similar angle, Pukale et al. has conducted in-depth pre-clinical examination of the use of LPN to treat psoriasis [44, 45, 83]. By evaluating the encapsulation of either clobetasol propionate, vitamin D3, or co-enzyme Q10 (CoQ10) in three separately published studies, the authors had provided a broad and detailed overview of the potential of LPN within this disease area [44, 45, 83]. Although drug permeation across the skin was only evaluated in the study with clobetasol propionate as the active component, findings from the study were extremely promising with the LPN formulation (17.87 ± 0.03 µg/cm^2^) having a 12-fold higher amount of drug present in viable epidermis and dermis layers of the skin as compared to a commercially available clobetasol propionate gel, Clobetamos™ (1.45 ± 0.61 µg/cm^2^) [83]. No detectable amounts of clobetasol propionate being found in the receptor medium of the ex vivo swiss albino mice skin permeation study and the plasma samples of the in vivo swiss albino mice (10—12 weeks old) systemic absorption model further solidified initial findings whereby LPN could improve permeation depth while limiting systemic absorption through the skin [83]. Across all three studies by this research group, using an imiquimod-induced psoriasis-like skin inflammation in Swiss albino mice model, the LPN formulations tested proved to have greater efficacy in than the plain gel tested without LPN embedded within the formulation as reflected by the Psoriasis Area and Severity Index (PASI) [44, 45, 83]. Through the findings of these individual studies, the potential of LPN to improve therapeutic efficacy of active compounds for the treatment of psoriasis by simply manipulating the controlled release, retention, and distribution within the active site has been highlighted.

Continuing on the theme of augmenting skin permeation, Keshari et al. investigated the feasibility of a developed LPN formulation in improving the therapeutic outcomes of Rapamycin (a PI3K/Akt/mTOR signalling pathway inhibitor), with a detailed study design centred around skin permeation [105]. Through a combination of an ex vivo skin permeation using Swiss albino mice skin study and in vivo skin permeation and retention using an IMQ-induced psoriasis in a Swiss albino mice (8—12 weeks) model, the authors created a comprehensive overview of the capabilities of their nanoparticulate system [105]. It was revealed that the LPN-based formulation had indeed produced significantly better mean skin penetration depth (tenfold higher) than the free drug alone, with data further supporting the bioadhesive nature of the formulation allowing the LPN-based formulation to retain at the site of active longer than the free drug alone as evidenced by the In Vivo Imaging System (IVIS) fluorescence signal over time [105]. Interestingly, the LPN-based formulation only displayed improved penetration depth and retention in diseased psoriatic skin, but a lesser extent was observed in healthy skin [105]. This evidence of targeted delivery could potentially lead to selectivity and reduction in adverse effects, as the drug would accumulate more in diseased skin but less in healthy skin. Based on this observed behaviour, the authors inferred the improved therapeutic activity of the LPN-formulation relative to the free drug as demonstrated by the PASI scoring and histopathology results, were likely a direct result of this [105].

Suzuki et al. had published a study on the use of LPN carrying TNF-α siRNA coupled with a photosensitiser (TPPS2a) which provides photochemical internalization (PCI) functionality to the system, optimising endosomal escape of the cargo within cells [80]. By using an ex vivo porcine skin model, the authors reported remarkably higher permeation and retention of TPPS2a and TNF-α siRNA in the skin relative to a TPPS2a solution [80]. Although the enhanced ability to permeate into deeper layers of the skin is highly advantageous, it can also be seen detrimental if the formulation readily allows for permeation across the entirety of the integument, potentially reaching the systemic circulation. This would cause unwanted systemic exposure to a localised skin disorder, highlighting the importance and precarity of developing such formulations whereby a balance must be struck to achieve optimal outcomes. Authors of this study had also shown improvements in psoriatic symptoms in an in vivo IMQ-induced psoriasis hairless mice model, which was attributed to the improved skin penetration and retention which was further evidenced by the TNF-α expression [80]. Findings from this study also determined statistically significantly lower TNF-α expression in the LPN-formulation treated groups (1.38-fold knockdown; *P* < 0.05) relative to the untreated diseased group, whereas the TPPS2a solution alone was not able to produce any significant effects [80]. Navigating drugs through the skin barrier is a notable challenge by itself, in the case of psoriasis, the added impediments by virtue of hyperproliferation of keratinocytes and abnormal skin thickening makes the task more daunting. Collectively, these studies have shown the extensive capabilities of LPN in overcoming these obstacles with a recurring theme of improved skin penetration depth and retention across almost each study, while being able to encapsulate a wide range of active compounds ranging from synthetic medicinal compounds and natural products to genetic material such as siRNA.

Overall, psoriasis has become one of the most heavily researched areas for the clinical applications of LPN among the common dermatological conditions. Collectively, the studies cover an impressive array of pharmacological cargos encompassing natural products, conventional therapeutic compounds, and genetic material. Incorporation of genetic material such as TNF-α siRNA together with a photosensitiser within an LPN to treat psoriasis provides a glimpse into the complexity which can be attained by this drug delivery system to achieve exceptionally narrow targeting of specific pharmacological pathways (Ref 48 same as the paragraph right above this one). The impressive repository of studies on LPN and psoriasis show tremendous promise of the application of this system for this particular disorder granted by the improvements in skin permeation and deposition, as well as stability and controlled release of the therapeutic cargo.

#### Cancer

In an effort to develop a targeted anti-melanoma pharmaceutical product, Silva et al. trialled the use of LPN functionalised with α-melanocyte stimulating hormone (α-MSH) carrying parvifloron D, a natural diterpene with non-selective cytotoxic activity [124]. Unfortunately, the authors were not able to identify significant differences in permeation through an in vitro permeation study employing a parallel artificial membrane permeability assay (PAMPA) between free parvifloron D and LPN-encapsulated parvifloron D [124]. However, this was likely not due to the behavioural differences between the two groups but instead the authors acknowledge a flaw in their experimental design whereby parvifloron D exhibited strong adsorption to the artificial membrane used [124]. Through a in vivo melanoma xenograft model using hairless SHO-SCID mice (42-years old), the authors had found that both free parvifloron D and LPN-encapsulated parvifloron D had produced extensive necrosis and haemorrhage post-treatment, without any significant reduction in tumour size [124]. Despite somewhat promising results from the in vitro cytotoxicity and cell internalisation assays, the same effects could not be observed when translated into an in vivo model, highlighting the challenge in translating drug delivery technology into a practical setting [124].

On the other hand, one study compared the use of cRGDyk, cyclic (Arignine-Glycine-Aspartic acid-D-Tyrosine-Lysine) peptide conjugated, polymeric (PLGA nanoparticles) and LPN-based formulations as a targeted photodynamic therapy (PDT) system, using ferrous chlorophyllin (Fe-CHL) as the photosensitiser (PS) [125]. As a result, the highly complex system exhibited substantial cytotoxic and cellular uptake effects when tested in vitro on melanoma cells (mouse melanoma cell line, B16.F10) [125]. This has been attributed to the singlet oxygen ^1^O_2_ production which is the central theme of the formulation designed which can produce localised damage to the melanoma cells [125]. However, the lack of data on non-melanoma cell lines raises uncertainty on the selectivity of the developed delivery system [125].

Barone et al. developed simvastatin-loaded chitosan-coated NLC in the form of hybrid nanostructured films as a co-adjuvant therapy for melanoma, studying the skin permeation, cytotoxic efficacy, and clinical safety [126]. In an ex vivo skin permeation study using newborn pig epidermis (~ 3 weeks old), the authors surprisingly found the use of chemical permeation enhancers (CPEs) was not favourable to improve skin permeation of simvastatin in the formulations [126]. Notably, the study was conducted without free simvastatin as a negative control, thus the exact performance of the formulation compared to the drug alone cannot be drawn [126]. In terms of the cytotoxic efficacy, there was no cytotoxic effect seen in both free simvastatin and the formulation within 3.125—100 μM against human keratinocyte (HaCaT) and fibroblast (COS-7) cell lines [126]. As for the melanoma cell lines, the formulation exhibited greater cytotoxic effects than free simvastatin in COLO-38 cell lines, with the greatest cytotoxicity observed when used in combination with limonene as a CPE [126]. The authors had also tested the safety of the developed formulation on 8 healthy human volunteers (both sexes, aged 30 ± 3 years) by evaluating skin irritation over 72 h [126]. The formulations tested did not exhibit significant skin irritancy, other than the formulation containing limonene as a CPE, based on measurements of induced erythema [126]. Based on TEWL measurements, all the formulations tested did not display any sign of irritancy [126].

LPN have also been studied in delivering conventional anticancer agents such dacarbazine (DCZ), a first-line antineoplastic agent indicated for use in treating melanoma [34]. In an ex vivo skin permeation study using a full thickness Wistar rat model, it was shown that the DCZ-loaded LPN formulation (80.71 ± 0.69%) had significantly greater permeation than DCZ alone (20.34 ± 1.74%) after 12 h of incubation [34]. Post-analysis of the data collected further revealed a drug flux of 15.93 ± 1.61 and 3.95 ± 0.89 μg·cm^2^·h^−1^ for the DCZ-loaded LPN formulation and DCZ alone groups, respectively [34]. When evaluating the in vitro cytotoxicity against B16F1 mouse melanoma cell lines, DCZ-loaded LPN (0.16 mg/mL) had a lower IC_50_ value compared to DCZ alone (0.49 mg/mL) [34].

In a separate study by Gonçalves et al., solid LPN were used as a delivery system, encapsulating glycoalkaloids, as a topical treatment strategy for melanoma [81]. By using an ex vivo porcine ear skin model, the authors found that the formulation produced effective disruption of the stratum corneum 2 h post-exposure to the treatment [81]. Ultimately, the formulation penetrated the skin achieving a depth of approximately 77 μm, while the free drug alone did not show any penetration across the skin barrier [81]. In vitro cytotoxicity tests revealed that the LPN formulation containing the glycoalkaloid displayed reduced toxicity human foreskin fibroblast cells (HFF-1) (IC_50_: 7.6 ± 0.3) while maintaining a similar toxicity to human cutaneous melanoma cells (SK-MEL-28) (IC_50_: 5.3 ± 0.2), with a selectivity index of 1.43 as compared to the glycoalkaloid alone (HFF-1 IC_50_: 2.6 ± 0.1; SK-MEL-28 IC_50_: 4.3 ± 0.2) with 0.60 as the selectivity index [81].

Collectively, the studies conducted to date mostly center around in vitro cytotoxicity models [34, 81, 124–126]. Although showing promising results, as shown in the study conducted by Silva et al., the translational aspect when moving into an in vivo animal model is difficult to predict, especially for a complex disease such as cancer. Absence of any encouraging in vivo animal model data within this area raises concerns for any potential clinical translation in the near future. At the current stage, applications of LPN for skin cancer still remain in its infancy requiring more time and effort to determine its future potential. Lack of direct comparisons between the separate polymeric and lipid components against the LPN formulation within the current literature also presents a challenge in determining whether a significant improvement is truly obtained by using LPN for skin cancer.

#### Pain

At the time of writing, two separate studies have been conducted to determine the potential use of LPN as a topical skin delivery system for lidocaine to improve its pharmaceutical profile as a local anaesthetic [106, 127]. Despite being a widely-used local anaesthetic, being a low molecular weight small molecular compound, lidocaine is rapidly absorbed and cleared from the skin leaving its effects short-lasting [106, 127]. Formulation-based strategies such as LPN present as a promising solution to this matter by improving retention time in the skin, offering longer-lasting activity. For this purpose, Wang et al. developed lidocaine-loaded LPN and evaluated the skin permeation and anaesthetic efficacy using an ex vivo Sprague–Dawley rat skin model and an in vivo tail flick latency (TFL) test, respectively [127]. The authors had found that lidocaine-loaded LPN outperformed both lidocaine-loaded liposomes and free lidocaine alone in terms of skin permeation, achieving a drug flux of 65.4 ± 3.1 μg/h/cm^2^ relative to the two latter groups, 40.6 ± 3.5 μg/h/cm^2^ (*P* < 0.05) and 29.3 ± 1.8 μg/h/cm^2^ (*P* < 0.05), respectively [127]. Furthermore, the in vivo TFL test revealed an increasing trend of local anaesthetic effect duration beginning from free lidocaine (peak effect lasting 10 min), followed by lidocaine-loaded liposomes (peak effect lasting 20 min), and lidocaine-loaded LPN (peak effect lasting 30 min) [127]. Authors attributed these findings to the polymeric core encapsulating lidocaine together with the lipid surface of the overall nanostructure [127]. Building on this, the lipid shell surface already provides enhanced retention, possibly due to improved permeation into the skin as reflected by the liposome group [128]. By adding a polymeric core structure, this enables the formulation to retain the drug within the system with fewer instances of drug leakage from the more fluid lipid layer, thus compounding their effects [19, 129]. Meanwhile, in a separate study, it was found through the use of an in vivo electrical stimulation test that lidocaine-loaded LPN produced a middle period of painlessness of 25 h as compared to lidocaine-loaded PLGA nanoparticles (20 h; *P* < 0.01) and lidocaine alone (1 h; *P* < 0.01). This time compared with a polymeric nanoparticle system, the authors argue that the improved effects are a virtue of the slower drug release profile of the formulation as reflected by their in vitro drug release studies.

Li et al. studied the use of LPN to encapsulate ropivacaine, another local anaesthetic similar to lidocaine [130]. Findings from their ex vivo skin permeation study had shown ropivacaine alone had produced rapid but limited permeation across the skin, peaking within the first few hours at a plateau of 344 ± 23 μg/cm^2^ [130]. On the other hand, ropivacaine-loaded LPN had produced extended effects, with gradual accumulation of drug permeation, peaking at 24 h with a plateau of 907 ± 35 μg/cm^2^ which is 2.6-fold higher (*P* < 0.05) than ropivacaine alone [130]. In terms of local anaesthesia effect, the authors used a TFL test in an in vivo rat model and an electrical stimulation test in an in vivo murine model [130]. In the TFL test, it was demonstrated that the anaesthetic effect of ropivacaine-loaded LPN lasted about 10 h as compared to ropivacaine alone, lasting only 1 h (*P* < 0.05) [130]. Similarly, the developed formulation sustained an analgesia duration lasting 36 h which is remarkably longer than the drug alone, achieving an effect lasting only 0.5 h post-application [130]. Aside from the previously discussed advantages of pairing lipids and polymers within a nanoparticle system in skin delivery, the formulation developed by these authors showed a biphasic release profile, providing a rapid initial drug release of 30% followed by a sustained release over 72 h [130]. This added property explains how the formulation was able to produce almost similar rapid effects as the drug alone while sustaining a longer duration of effect.

The use of LPN were also compared against NLC, both modified with a transcriptional transactivator peptide, in co-delivering dexmedetomidine and levobupivacaine, for local anaesthesia [131]. In this case, the modified-NLC demonstrated superior activity skin permeation effect, but only for the first 24 h [131]. After the initial 24 h up to 48 h, the LPN produced greater cumulative permeation [131]. Once again, this echoes the results from the previously discussed studies, whereby the use of a lipid and polymer hybrid system consistently achieves superior extended-release properties compared to lipid- or polymer-based systems alone. Reflecting the skin permeation profiles, the longest anaesthetic effect observed in this study was in the LPN group, 60 h, followed by the modified-NLC group, 48 h, which both outperformed their non-modified counterparts which only achieved 12 h and 8 h of effects, respectively [131]. In an in vivo nociception study through a hot plate test, the authors also showed better sustained pain threshold, greatest in the LPN group followed by the modified-NLC group [131]. Overall, the inferences generated based on the findings from this study largely surrounds the improvements observed in skin permeation by using a modified-LPN formulation [131].

Data from two separate studies by Wang et al. and Li et al. provided clear insight into the improved therapeutic outcomes achievable using LPN formulations compared to either liposomes or NLC on their own [127, 131]. In both these studies, incorporation of a polymeric component in a LPN-based system provided improved skin permeation which subsequently translated into better analgesic or anaesthetic efficacy [127, 131]. Generally, the consensus for LPN-based formulations and pain can be seen in a similar light, with studies repeatedly showing the improved skin permeation achieved by their respective LPN formulations followed by improved analgesia or anaesthesia.

#### Cosmetic

Nanotechnology has long since played an instrumental role in the cosmetics industry, with various applications and uses [5]. In fact, the cosmetics industry is a fine example of successful translation of academic research into industrial areas whereby the impact of research directly reaches the public [5]. From perfumes and colognes to skincare and hygiene products, the use of nanotechnology is boundless within this field, contributing to its growth as a leading market with a 2022 retail sales value of 97 billion euros in just the United States (US) alone [5, 132].

Holding onto this sentiment, a considerable number of studies have implemented the use of LPN to benefit from their many advantages within this area of research. One of such studies by Zhao et al. experimented with the use of LPN-loaded dissolving microneedle patches for miR-218, a microRNA which promotes dermal papilla cell proliferation, delivery to promote hair regrowth [107]. Findings from this study show that LPN was successfully incorporated into microneedles, serving as a viable strategy, improving the maximum amount of microRNA absorbed into the skin reaching 1.33 ± 0.24 μg/mL as compared to 0.38 ± 0.06 μg/mL found in the LPN-gel group [107]. Based on histological analysis, it was also clear that the hair regrowth in the LPN-microneedle group was significantly better than the LPN-gel group, achieving 27.9 ± 9.4 μm and 19.3 ± 2.2 μm of hair thickness, respectively, after 13 days [107]. Continuing on the topic of hair regrowth, Das et al. investigated the use of LPN for the transfollicular delivery of quercetin to treat androgenic alopecia [133]. The authors qualitatively demonstrated through fluorescence microscopy that the LPN produced increased follicular uptake in an in vivo Sprague–Dawley rat model as compared to PLGA nanoparticles, eliciting the key advantage of lipids within the formulation to favor the sebum-filled environment of hair follicles [133]. Histological evaluation and hair follicle density measurements further proved healthier hair regrowth with maximum densities reaching 2.7 ± 0.3, 2.4 ± 0.35, and 3.8 ± 0.15 number/mm for the LPN-treated group, PLGA nanoparticle-treated group, and marketed minoxidil product, respectively [133]. Although performing poorer to the marketed product in terms of hair density, application of LPN may have a role in improving the quality of hair regrowth according to histological analysis indicating presence of most hair follicles present longer, denser, deeper, and less necrosed [133]. In terms of transfollicular delivery, certain fundamental barriers such as size selectivity, sebum, and hair growth cycles present a challenge [134]. Existing within the nanometer scale, size selectivity imposes no challenge for LPN-based formulation or PLGA nanoparticles used in this study [134]. The highly lipophilic sebum presents as a significant barrier to PLGA nanoparticles which can be surmounted to an extend by incorporating a lipid component through an LPN formulation [134]. The limited improvements observed in this study could be due to the additional challenge of hair growth cycles which further impedes effective delivery of the active compound to the site of action, thus limiting its therapeutic effects.

Pena-Rodriguez et al. investigated the use of LPN in encapsulating dexamethasone across two separate studies published in the same year, primarily focusing on the nanoparticle and drug distribution within the skin [35, 36]. Through immunofluorescence techniques and confocal laser scanning microscopy (CLSM), the authors found that incorporation of dexamethasone into LPN increased the accumulation of the drug within hair follicles [35]. These findings resonate with the earlier findings by Das et al. with both groups of investigators citing the additional lipophilic nature of the lipid component of the system as the culprit for this observation [35, 133]. In an ex vivo pig skin permeation test, Pena-Rodriguez et al. had also found improved permeation using dexamethasone-loaded LPN suspension compared to dexamethasone hydrogel and dexamethasone-loaded LPN hydrogel [36]. Authors cite the slower release rate of the hydrogel formulations as a limiting factor contributing to this, once again highlighting the differences in effectiveness from using different macroscale drug delivery systems as highlighted by Zhao et al. in their study [36, 107].

Two separate studies by different authors published in the same year had a look at LPN incorporating chitosan as the polymeric component of the system to encapsulate phytochemicals [62, 78]. Abosabaa et al. [78] employed a chitosan and lecithin based LPN for the delivery of green tea extract for anti-cellulite effects. In terms of skin permeation and deposition, only caffeine was found to completely permeate the skin barrier in an ex vivo rat skin permeation model [78]. Each component of the green tea extract retained within the skin in varying degrees, caffeine, 69.25 ± 3.63%; catechin, 72.28 ± 6.41%; epicatechin, 93.98 ± 5.48%; epigallocatechingallate, 28.21 ± 2.07%, the remaining amounts unretained reamined on the skin surface [78]. These results act as evidence that the individual physicochemical properties of each compound still plays a crucial role in the distribution within the skin despite being encapsulated within a LPN-based system [78]. Similarly, a study by Castro et al. reported the use of chitosan-coated lipid vesicles as a LPN system to deliver octyl methoxycinnamate as a photoprotective agent [62]. Greater sun protection factor (SPF) values were seen in the LPN-based formulation (SPF 9.7—10.3) as compared to the compound alone (SPF 7.3) [62]. Authors attributed this finding to the slower release profile exhibited by the LPN formulation [62].

One study has developed a MCLV as a complex LPN-based system encapsulated ATRL for skincare purposes [40]. By using an in vitro artificial skin model (EpiDerm™), authors demonstrated negligible activity for free ATRL, but profound increase in collagen gene (COL1A1) expression (*P* < 0.05) while significantly downregulating matrix metalloproteinase (MMP) gene expression (*P* < 0.001) [40]. Improved penetration was ascribed as the reason for this observation, however, a lack of penetration data was presented in the study to provide evidence for this claim [40].

## Gaps in the current research and limitations

By and large, LPN have emerged as a substantial contender in the field of dermatological drug delivery, demonstrating superior skin permeation, controlled drug release, and therapeutic efficacy compared to the individual counterparts (e.g. NLC, liposomes, PBN). However, despite the various advantages offered by LPN, there are still a number of hurdles to be overcome before achieving clinical translation and widespread adoption.

One of the fundamental considerations in this complex nanoparticle system is the integration of strengths between both LBN and PBN, complementing each other in a single drug delivery system. However, while succeeding the boons from both LBN and PBN, they may potentially inherit their respective limitations. This becomes particularly evident with regards to the stability and polydispersity of these hybrid systems. Liposomes, a well-established vesicular LBN, are inherently unstable; combining such a system with PBN may compromise the stability of an otherwise robust polymeric system. This review of recent LPN relevant studies in dermatological disorders revealed more than half of the literature reporting at least one issue of physicochemical instability. Generally, this presents a significant barrier in obtaining approval from regulatory authorities as pharmaceutical formulations typically require stringent stability testing to demonstrate an acceptable shelf-life quality. Likewise, this also poses a significant challenge in maintaining fundamental formulation properties while delivering API across the skin barrier.

Furthermore, SLN and NLC are favoured for the simplicity in formulation methodology in regards to the lack of a need to use organic solvents, yet the polymeric component in LPN often necessitates its use, reintroducing toxicity-related challenges [135]. Adding to this, the simple, cost-effective, and scalable formulation process of LBN become absent when formulating LPN which instead require multiple formulation steps making them more labour intensive and costly. Based on the current scope of this review, only one study has demonstrated the feasibility of scaling up LPN production, suggesting that while manufacturing complexity remains a considerable challenge, it may be a surmountable one with continued research.

Throughout this review, it has been identified that several novel types of LPN have not been widely discussed in recent review articles. Most contemporary reviews primarily focus on PCLSHN, biomimetic membrane-coated LPN, core–shell type hollow lipid-polymeric hybrid nanoparticles, polymeric-cage nanobins, and monolithic LPN. In contrast, emerging formulations such as solid LPN, despite exhibiting structural similarities to monolithic LPN, often remain insufficiently characterised and validated. The complexity of this particular nanoparticle formulation leads to an inevitable challenge in systematic classification, especially considering the rapid evolution in the field. This matter becomes vexing for researchers seeking to navigate the complex field. This matter poses a significant challenge for researchers seeking to navigate the complex field. To enhance clarity, reproducibility, and scientific rigor in LPN research, it is essential to adopt comprehensive characterisation approaches that incorporate both qualitative imaging techniques and complementary analytical methods.

A similar issue detected is with regards to the methodological aspects of LPN formulation. Current literature has been found discussing classical formulation strategies, mostly involving two-step and one-step nanoprecipitation methods, as well as emulsification-solvent evaporation methods (single and double emulsion). However, it has been noted that recently developed LPN fabrication methods which were encountered during this review could potentially be overlooked. A potential cause for this is the inconsistency in method descriptions, making direct comparisons difficult to perceive, or the lack of distinction in naming conventions, leading to the unintended omission in broader methodological discussions. Considering the various methods discussed, the classification of two-step and one-step approaches in LPN formulation remains inherently misleading due to the oversimplification of a complex process. This arises as descriptions of these methods typically disregard the preparation of the polymeric phase in the two-step approach and the necessity of separately preparing polymeric and lipid phases in the one-step approach. Moreover, lengthy solvent evaporation and centrifugal-based purification steps extend the protocol beyond just one or two steps. This misrepresentation has critical implications for clinical translation because pharmaceutical manufacturers prioritise simplicity and streamlined processes for large-scale production. Therefore, one must carefully understand the complexity of LPN formulation to avoid misinformation by the oversimplified classification.

In terms of topical dermal drug delivery to achieve localised effects in diseases relating to the integumentary system, LPN have shown to perform well in certain areas but have been lacking representation in disease areas such as atopic dermatitis, one of the most common skin conditions as well as wound healing and skin cancer. The limited amount of research conducted in these areas limits the understanding of the capabilities of LPN and their interactions with the pathological elements surrounding these diseases.

The variety of skin models used throughout the studies gathered in this review ranging from mice and rats to pigs, with one study reporting the use of goat ear skin, leads to challenges in making direct comparisons between studies [136]. This further adds difficulty in extrapolating the results from these models into a clinical setting to predict results in humans upon clinical translation. A number of studies in this review has also reported exceptional permeating effects of the developed LPN formulations, permeating across the entire skin barrier. Practically, this may be a detriment to LPN formulations as the targeted skin-disorders require localised delivery on the skin. Permeation of API across the skin barrier may lead to inadvertent systemic absorption, raising issues of systemic toxicity.

## Future direction and perspective

From a broader perspective, LPN present both opportunities and challenges in dermal drug delivery. The pharmaceutical industry tends to be risk-averse when adopting new technologies; unless they offer clear advantages in efficacy, safety, and cost-effectiveness. This highlights the need to refine LPN formulations to balance their benefits with manufacturability. Encouragingly, ongoing research is heading in a direction focusing on practical, scalable, and regulatory-compliant approaches. The versatile combinations of LPN due to their ample choices of building blocks as well as various structural permutations provide endless possibilities for optimisation. By tuning the nanoparticle characteristics through this way, varied therapeutic outcomes of the desired formulation can be achieved as well.

Insights into the technological advancement trends particularly with regards to commercial translation of academic research, can be gained through analysing patent trends. A vast majority of patents for LPN have been found to be in treating complex diseases such as cancer. Logically, the complexity of LPN-based formulations allow for precise fine-tuning and extensive modifications allowing for the delivery into complex drug delivery environments such as cancer tumours. To date, one patent has been applied and granted in the year 2020 (US 10,850,246 B2) titled Method for preparing pH dependent ultra small polymeric nanoparticles for topical and/or transdermal delivery. The patent describes a novel methodology employing nanoprecipitation and layer-by-layer nanodeposition to formulate ultra-small polymeric-lipidic delivery nanoparticles. The invention claims to produce pH controlled drug release with small sizes of 5—25 nm and the ability to penetrate the skin strata to combat a variety of skin disorders. Despite a considerable amount of research publications, the lack of patents being filed or granted for LPN and clinical trials in dermal drug delivery highlights a significant blind spot in the field where researchers use the opportunity to bring their research into clinical translation.

Ultimately, the future of LPN-based research relies on addressing the fundamental challenges raised before. A promising research direction is the strategic selection of the right lipid-polymer combinations that complement each other in drug delivery performance, stability, and scalability. Rather than merely combining existing systems into a hybrid platform, rational formulation design prioritising retention of the beneficial properties of LBN and PBN while mitigating their drawbacks must govern the decision-making process of researchers in this field.

Despite the challenges, the pursuit of LPN technology presents an exciting area in drug delivery sciences. With the right formulation strategy, LPN offer a unique convergence of lipid- and polymer-based advantages, enabling precise drug release control, improved skin permeation, and enhanced therapeutic outcomes. Their capacity to co-encapsulate multiple API makes them particularly valuable for complex treatments, skin disorders normally requiring a cocktail or combination of multiple drugs. Continued interdisciplinary collaboration between academia and industry is essential to develop more refined, stable, and cost-effective LPN systems, paving the way for next-generation nanomedicine, expanding treatment possibilities and addressing healthcare needs.

## Conclusion

Overall, LPN represent a highly promising platform for topical dermal or cutaneous drug delivery. By integrating the complementary benefits of both lipid- and polymer-based systems, LPN offer remarkable versatility, allowing for extensive modifications that can be tailored to a wide spectrum of skin disorders. Despite these advantages, several critical limitations, ranging from the fundamental formulation parameters to challenges in clinical translation – remain and warrant focused investigation. Addressing these gaps is essential to unlocking the full therapeutic potential of LPN. Future research should prioritise the standardization of classification schemes and formulation methodologies to enhance reproducibility, comparability and ultimately, the clinical viability of LBN-based drug delivery systems for dermatological applications.

## Data Availability

More information regarding the data presented in this review will be available upon request.
